# Tetraspanin (TSP-17) Protects Dopaminergic Neurons against 6-OHDA-Induced Neurodegeneration in *C. elegans*

**DOI:** 10.1371/journal.pgen.1004767

**Published:** 2014-12-04

**Authors:** Neda Masoudi, Pablo Ibanez-Cruceyra, Sarah-Lena Offenburger, Alexander Holmes, Anton Gartner

**Affiliations:** 1 Centre for Gene Regulation and Expression, University of Dundee, Dow Street, Dundee, United Kingdom; The University of Alabama, United States of America

## Abstract

Parkinson's disease (PD), the second most prevalent neurodegenerative disease after Alzheimer's disease, is linked to the gradual loss of dopaminergic neurons in the substantia nigra. Disease loci causing hereditary forms of PD are known, but most cases are attributable to a combination of genetic and environmental risk factors. Increased incidence of PD is associated with rural living and pesticide exposure, and dopaminergic neurodegeneration can be triggered by neurotoxins such as 6-hydroxydopamine (6-OHDA). In *C. elegans*, this drug is taken up by the presynaptic dopamine reuptake transporter (DAT-1) and causes selective death of the eight dopaminergic neurons of the adult hermaphrodite. Using a forward genetic approach to find genes that protect against 6-OHDA-mediated neurodegeneration, we identified *tsp-17*, which encodes a member of the tetraspanin family of membrane proteins. We show that TSP-17 is expressed in dopaminergic neurons and provide genetic, pharmacological and biochemical evidence that it inhibits DAT-1, thus leading to increased 6-OHDA uptake in *tsp-17* loss-of-function mutants. TSP-17 also protects against toxicity conferred by excessive intracellular dopamine. We provide genetic and biochemical evidence that TSP-17 acts partly via the DOP-2 dopamine receptor to negatively regulate DAT-1. *tsp-17* mutants also have subtle behavioral phenotypes, some of which are conferred by aberrant dopamine signaling. Incubating mutant worms in liquid medium leads to swimming-induced paralysis. In the L1 larval stage, this phenotype is linked to lethality and cannot be rescued by a *dop-3* null mutant. In contrast, mild paralysis occurring in the L4 larval stage is suppressed by *dop-3*, suggesting defects in dopaminergic signaling. In summary, we show that TSP-17 protects against neurodegeneration and has a role in modulating behaviors linked to dopamine signaling.

## Author Summary

Parkinson's disease (PD) is characterized by the progressive loss of dopaminergic neurons. While hereditary forms are known, most cases are attributable to a combination of genetic and environmental risk factors. In PD models, dopaminergic neurodegeneration can be triggered by neurotoxins such as 6-hydroxydopamine (6-OHDA). This drug, which is taken up by the presynaptic dopamine reuptake transporter (DAT-1), also causes the selective death of *C. elegans* dopaminergic neurons. We found that TSP-17, a member of the tetraspanin family of membrane proteins, protects dopaminergic neurons from 6-OHDA-induced degeneration. We provide evidence that TSP-17 inhibits the *C. elegans* dopamine transporter DAT-1, leading to increased neuronal 6-OHDA uptake in *tsp-17* mutants. TSP-17 also protects against toxicity conferred by excessive intracellular dopamine. TSP-17 interacts with the DOP-2 dopamine receptor, possibly as part of a pathway that negatively regulates DAT-1. *tsp-17* mutants have subtle behavioral phenotypes that are partly conferred by aberrant dopamine signaling. In summary, we have used *C. elegans* genetics to model key aspects of PD.

## Introduction

Parkinson's Disease (PD) is the second most common neurodegenerative disease, after Alzheimer's disease, and affects ∼2% of the population aged over 65 years. Loss of dopaminergic neurons is a pathological hallmark of PD [1], [2] and aspects of this neurodegeneration have been modeled in *C. elegans* [3], [4]. The etiology of PD is largely unknown and its heritability is generally rather low; however ∼5–10% of cases are associated with monogenetically inherited mutations [5]. Approximately 15 disease loci are known, most of which are conserved in *C. elegans* [6], [7]. The vast majority of PD cases are ‘sporadic’ with no clear family history. Besides aging, epidemiological studies have shown risk factors for ‘sporadic’ PD to include a long-term history of rural living, farming, well-water drinking and pesticide exposure. The most extreme examples of toxin-induced PD-like symptoms were linked to the accidental exposure to MPTP (*N*-methyl-4-phenyl-1,2,3,6-tetrahydropyridine). Similar to sporadic PD cases, PD-like symptoms resulting from MPTP exposure could be alleviated by administration of the dopamine precursor L-3,4-dihydrooxyphenylalanine (L-DOPA) [8]. Exposure to pesticides such as paraquat and rotenone has also been implicated in PD development [9]. The disease is therefore thought to be triggered by a combination of environmental factors and genetic susceptibility [5].

MPTP, paraquat and rotenone all block the mitochondrial electron transport chain, leading to oxidative damage [10], and have been extensively used to model PD neurodegeneration. 6-Hydroxydopamine (6-OHDA), an oxidation product of dopamine, is another neurotoxin widely used in mammalian PD models to induce the specific degeneration of dopaminergic neurons [11]. 6-OHDA was initially identified as a metabolite of dopamine [12], and there is some evidence that 6-OHDA exposure might be linked to PD. 6-OHDA was also identified as a naturally occurring amine in human urine, and has been detected at higher concentrations in PD patients [13]. Furthermore, high 6-OHDA levels were found in postmortem brain samples from PD patients [14]. It has been reported that 6-OHDA interaction with oxygen results in the production of reactive oxygen species (ROS), which in turn trigger free radical-mediated neuronal degeneration [2], [12]. Other dopamine metabolites may also cause oxidative damage [15]. Nevertheless, the mechanism by which 6-OHDA induces neuronal degeneration remains largely unknown [16].

Although there is no treatment to prevent or halt neuronal loss, L-DOPA administration is still one of the most effective treatments for alleviating PD symptoms [17], [18]. However, the effectiveness of L-DOPA declines over time. Prolonged L-DOPA treatment is also potentially neurotoxic [11], [15]. Although not confirmed in a large longitudinal study of L-DOPA use in PD patients (ELLDOPA trial), this nevertheless remains a major concern [19].

*C. elegans* has been used as a model to study the structure and function of the nervous system, which in hermaphrodite worms consists of 302 neurons [20], [21]. *C. elegans* dopaminergic neurons are functionally related to those of humans. The genes driving the biochemical processes involved in dopamine metabolism (as well as most PD-associated loci) [6] are also highly conserved in worms [22]. Dopaminergic neurons can be readily visualized in vivo using appropriate GFP markers. Analogous to vertebrate systems, dopaminergic neurons undergo neurodegeneration upon treatment with 6-OHDA. It has been shown that 6-OHDA can enter dopaminergic neurons through the DAT-1 dopamine transporter and thus trigger their degeneration [3]. The exact type of cellular death that occurs following 6-OHDA intoxication is unknown. Electron microscopy has shown apoptotic-like condensed chromatin structures in dying neurons, suggesting that 6-OHDA induces apoptosis. However, 6-OHDA-induced neurodegeneration in *C. elegans* is independent of CED-4/Apaf1 and CED-3/caspase, two components of the core apoptotic machinery [3]. In an independent study, inactivation of *C. elegans* autophagy genes partially suppressed 6-OHDA-induced dopaminergic death, suggesting that autophagy might also be involved in this process [23].

During synaptic transmission most of the released dopamine is transported back into the presynaptic terminal by the dopamine reuptake transporter (DAT1) (for a review, see [24]. Therefore, activity of this transporter affects the duration and extent of dopamine signaling. Mammalian cell experiments led to the identification of several proteins that interact with DAT1 to modulate its activity, cell surface expression and trafficking. These include protein kinase C, dopamine D2 receptors (discussed below), SNCA and parkin [25]–[28]. The physiological actions of dopamine are mediated by conserved seven-transmembrane dopamine receptors, designated D1–5. Dopamine receptors are coupled to guanosine triphosphate-binding proteins (G proteins) and are classified into D1 or D2 type dopamine receptors based on their antagonistic effect on adenylyl cyclase activity [29], [30]. D1 dopamine receptors, DOP-1 in worms, are solely found in postsynaptic dopamine-receptive cells, whereas in *C. elegans* the D2 type receptors DOP-2 and DOP-3 are expressed pre and postsynaptically, respectively [31]–[33].

In vertebrates, the dopamine system plays a crucial role in regulating movement, reward and cognition. Dopamine-deficient newborn mice die as a result of severe motor impairments [34], [35]. In contrast, *C. elegans* mutants defective in dopamine synthesis are viable, thus facilitating investigations into dopamine-mediated behavior in these animals. Dopaminergic neurons in *C. elegans* are required for specific, well-described and quantifiable behaviors, often associated with locomotion and feeding. For instance, the basal slowing response allows worms to reduce their speed when encountering a bacterial lawn, which is their food source [36]. Another behavior mediated by dopamine signaling is referred to as “swimming-induced paralysis” (SWIP): *dat-1*-deficient worms exhibit rapid paralysis in liquid, unlike wild-type controls [37].

Using an unbiased forward genetic approach we identified *tsp-17* as a gene that protects dopaminergic neurons from 6–OHDA-mediated neurodegeneration. We provide evidence that TSP-17 regulates DAT-1 transporter activity. Furthermore, our results suggest that DAT-1 regulation by TSP-17 is partly mediated by D2 dopamine receptors.

## Results

**Figure 1:**
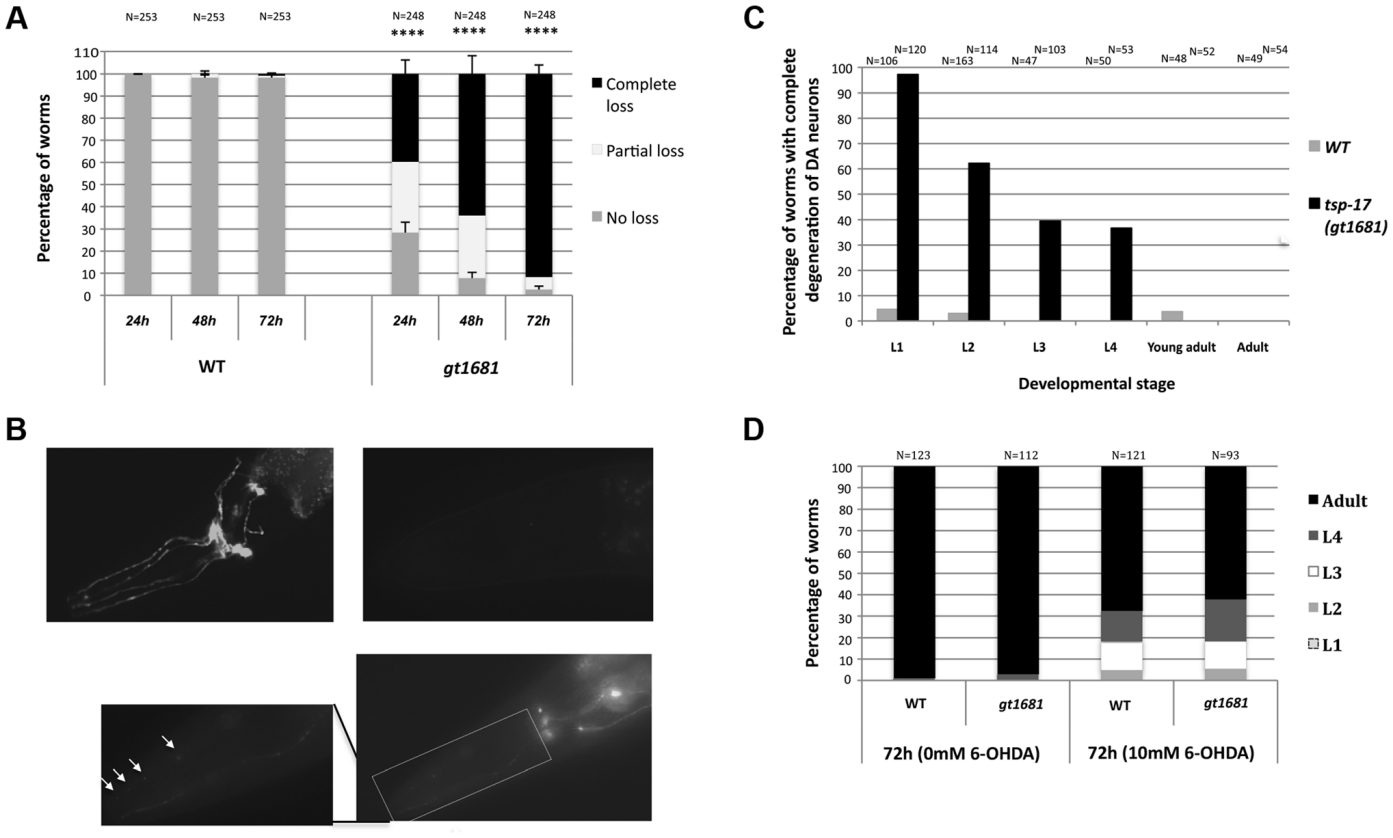
*gt1681* mutants are hypersensitive to 6-OHDA. **A**. The extent of dopaminergic degeneration is indicated for wild-type and *gt1681* mutant worms after intoxication with 10 mM 6-OHDA. Neurodegeneration of L1 worms was scored after 24, 48 and 72 h as described in Materials and Methods, and categorized as “complete loss,” “partial loss” or “no loss” phenotypes (labeled black, white and gray, respectively). Asterisks represent statistical significance of differences from wild-type (****p<0.00001). **B**. Representative images showing progressive stages of dopaminergic neurodegeneration. Absence of degeneration in wild-type (upper left panel) and complete degeneration in *gt1681* mutant worms 72 h post 6-OHDA intoxication (upper right panel, complete degeneration); lower panel and inset are examples of partial degeneration *in gt1881*. Arrows indicate ‘blebs in degenerating neurons. **C**. Extent of neurodegeneration at various developmental stages in wild-type and *gt1681* mutant worms 72 h post 6-OHDA intoxication. **D**. In wild-type and *gt1681* worms, development is equally retarded following treatment with 6-OHDA. Progression to various developmental stages was scored once 95% of untreated worms reached adulthood.

In order to find genes that protect dopaminergic neurons, we performed a genetic screen for mutants conferring hypersensitivity to 6-OHDA. By adapting procedures initially established by Nass et al. [3] and using the same *pdat-1::GFP* reporter that highlights dopaminergic neurons, we screened ∼2500 F2 ethyl methanesulfonate (EMS)-mutagenized worms at the L1 developmental stage by incubating with 10 mM 6-OHDA for 1 h. This procedure, which is based on reduced, altered, or absent *pdat-1::GFP* expression, does not lead to neurodegeneration in >95% of wild-type worms, thus allowing the identification of mutants conferring hypersensitivity to 6-OHDA. Of the initial five mutant candidates, only *gt1681* maintained a strong hypersensitive phenotype upon backcrossing (Figure 1A, Figure S1). 6-OHDA-induced degeneration of both wild-type and *gt1681* neurons exhibits the same morphological features and pattern of degeneration initially described by Nass et al. [3]. Axonal blebbing becomes apparent (Figure 1B, inset, arrows) a feature also consistent with morphological changes previously observed by electron microscopy. Worms were scored 24, 48 and 72 h after intoxication. Neurons were lost in less than 10% of wild-type worms after 72 h. In contrast, all dopaminergic neurons were lost in ∼40% of *gt1681* worms and partial dopaminergic loss was observed in an additional ∼30% of mutant worms after only 24 h (Figure 1A). The extent of neurodegeneration was further increased 72 h after intoxication, with ∼90% of worms displaying total dopaminergic loss at the adult stage (Figure 1A). Enhanced neurodegeneration in the *gt1681* background, albeit to a lesser extent, also occurred in L2, L3 and L4 larvae treated with 6-OHDA; no such enhancement was seen in adults (Figure 1C). To exclude the possibility that neurodegeneration might be caused by increased net 6-OHDA uptake at the organismal level, we took advantage of the partial growth retardation conferred by 6-OHDA treatment. By scoring for progression to ensuing developmental stages, we found the growth of wild-type and *gt1681* worms to be similarly retarded upon toxin treatment, suggesting that *gt1681* specifically affects dopaminergic neurons (Figure 1D).

**Figure 2:**
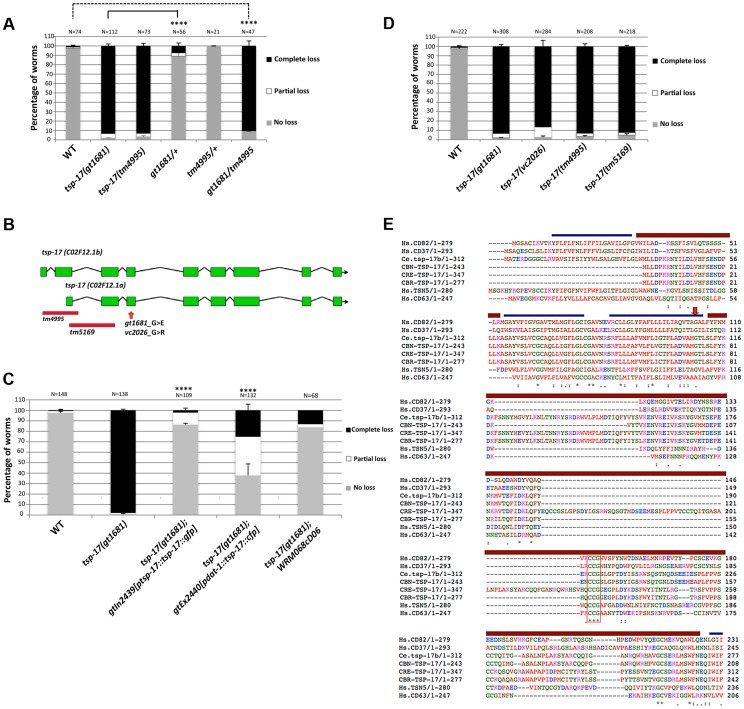
The TSP-17 tetraspanin family member protects dopaminergic neurons from 6-OHDA. **A**. Extent of neurodegeneration in heterozygous and trans-heterozygous worms 72 h post 6-OHDA intoxication. **B**. Schematic gene model of the two isoforms of *tsp-17*. Alleles used in this study are indicated. **C**. Complementation of *tsp-17* expressed under its own promoter (3^rd^ column strain TG2439) and under the *dat-1* promoter (4^th^ column, strain TG2440). Data presented is from scoring the extent of neurodegeneration 72 h post 6-OHDA intoxication. Asterisks represent statistical significance of differences between *tsp-17* and the rescuing lines (****p<0.0001). **D**. 6-OHDA hypersensitivity conferred by various *tsp-17* alleles. **E**. Alignment of nematode TSP-17 to the most closely related human tetraspanins. Blue bars indicate transmembrane domains and brown bars designate extracellular loops (EC1 and EC2). The arrow indicates amino acid G109, which is mutated in the *C. elegans gt1681* and *vc2026* mutants. The red box indicates the CCG motif in the EC2, which is highly conserved throughout the tetraspanin protein family. Hs, *Homo sapiens*; Ce, *Caenorhabditis elegans*; Cbn, *C. brenneri*; Cre, *C. remanei*; Cbr, *C. briggsae*.

The *gt1681* mutant is recessive in hermaphrodites (Figure 2A). Genetic linkage was established by single nucleotide polymorphism (SNP) mapping, which placed *gt1681* on the left arm of the X chromosome. Using *unc-20* and *lon-2* genetic markers to perform three-factor mapping, the locus was further refined to ∼10 map units. A cross between an *unc-20 gt1681 lon-2* triple mutant and the CB4856 “Hawaii” mapping strain enabled us to assess the position of single recombination events relative to *gt1681*. This analysis localized *gt1681* to an interval between nucleotides 3,659,480 and 3,737,466 on the physical map. In parallel, next generation sequencing revealed a single exonic mutation within this interval, leading to a guanine to adenine substitution in the C02F12.1 open reading frame and resulting in a glycine to glutamic acid change at position 109 of the encoded protein (Figure 2B). C02F12.1 encodes a tetraspanin family, integral membrane protein called TSP-17 (see below). Rescue of the phenotype by a fosmid (WRM0626aC02) encompassing *tsp-17* and by a *tsp-17*-encoding transgene (Figure 2C) provides further evidence that *gt1681* confers 6-OHDA hypersensitivity. Hypersensitivity is also conferred by the *vc2026* allele, a substitution obtained via the Million Mutation Project [38] that results in a glycine to arginine change at position 109 (Figure 2B, 2D). Finally, two deletion alleles, generously provided by the Japanese Knockout Consortium, affecting the first exons of *tsp-17* also confer hypersensitivity to 6-OHDA-mediated neurotoxicity (Figure 2B, 2D) as does the trans-heterozygous *gt1681/tm4995* mutant combination (Figure 2A).

Tetraspanins constitute a large protein family, with 30 and 21 members encoded in the human and *C. elegans* genomes, respectively [39]–[41]. Most tetraspanins have not been functionally characterized. In vertebrates, tetraspanins are suggested to be involved in cell–cell fusion, cell adhesion, cell motility and tumor metastasis [42]. In *C. elegans*, TSP-12 is involved in modulating Notch signaling, and specific hypodermal TSP-15 expression is required to mediate covalent tyrosine–tyrosine cross-linking during cuticle formation [43], [44]. *C. elegans tsp-17* is predicted to encode two isoforms. The large isoform, C02F12.1b, encodes a 312 amino acid protein containing four TM domains. The short isoform, C02F12.1a, encodes a 243 amino acid protein that, unlike typical tetraspanins, contains only three transmembrane domains and does not have an intracellular N-terminus. The amino acid change at position 109 in *gt1681* affects a highly conserved residue in the third transmembrane domain of the long isoform (Figure 2B, 2E). We confirmed expression of mRNAs encoding for both isoforms, and verified the predicted intron–exon structure (Figure 2B). Using BLAST protein analysis of *C. elegans* TSP-17, we found the most likely human orthologs of TSP-17 to be CD63, Tspan5 and CD82 (Figure 2E). A previous phylogenetic analysis placed TSP-17 within the human CD82 subfamily [45]. However, our attempts to firmly establish an orthologous relationship between TSP-17 and a single human tetraspanin or a distinct subfamily of human tetraspanins were unsuccessful. Our phylogenetic analysis included all tetraspanins from several nematodes, arthropods, cnidarians and chordates (Figure S2). We speculate that the rapid evolution of this protein family, as often occurs with membrane proteins, compromised our ability to firmly identify a human ortholog of *C. elegans* TSP-17.

**Figure 3:**
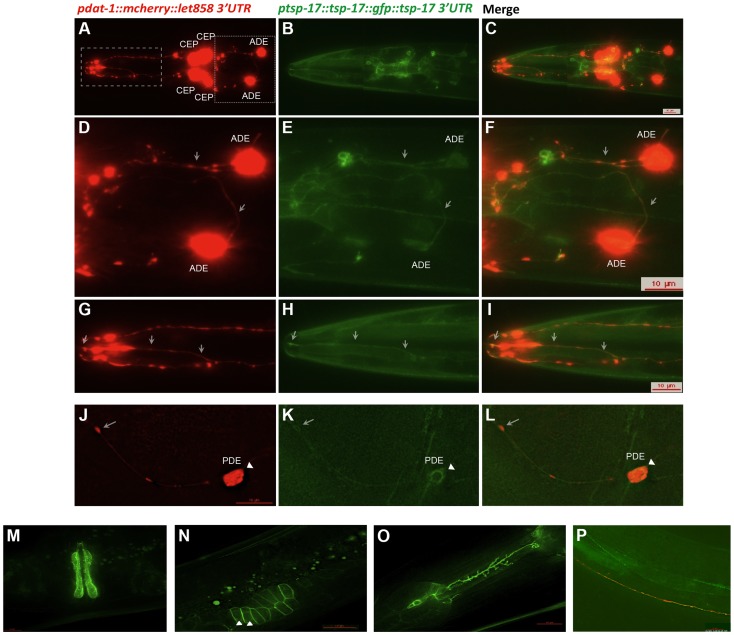
TSP-17::GFP expression. Analysis of the TG2439 strain containing dopaminergic neurons labeled by mCherry and *tsp-17* C-terminally fused to GFP and driven by its own promoter. **A, D, G, J**. Dopaminergic neurons expressing the mCherry marker. Neurons are indicated. White arrows highlight dendrites and axons. **B, E. H, K**. Expression of TSP-17::GFP. **C, F, I, L**. Merged images. White arrows highlight dendrites and axons. **K, L, N**. The arrow-heads indicate TSP-17::GFP signal enrichment around the nucleus. Expression in the vulva (**M**), the spermatheca (**N**), a NSM neuron (**O**), and in body wall muscle cells (**P**).

To assess the TSP-17 expression pattern, we used biolistic bombardment to generate transgenic worms (TG2439) expressing a *tsp-17::GFP* gene fusion (NM001) under the control of its own promoter and 3′UTR. A *dat-1 (promoter)::mCherry* fusion (PBI001) was co-bombarded to mark dopaminergic neurons. The *tsp-17::GFP* gene fusion largely suppressed the hypersensitivity phenotype conferred by *tsp-17*, thus confirming its functionality (Figure 2C, bar 3). Importantly, fusion protein expression was observed in all dopaminergic neurons: it was uniform along axons and dendrites of both dorsal and ventral pairs of CEP neurons, as well as in ADE neurons (Figure 3A–I, arrows indicate axons and dendrites) and in the posterior PDE neurons. Within the cell body, the TSP-17::GFP fusion seems to be excluded from the nucleus, a pattern that is more evident in a “close-up” image of a PDE neuron, where the signal appears to form a ring-like structure around the nucleus (Figure 3J–L arrowheads). mCherry aggregates (which are not linked to neurodegeneration) form dot-like structures in dendrites and axons (arrows), and the surrounding TSP-17 fluorescent signal suggests plasma membrane expression (arrow, Figure 3K). TSP-17 enrichment at the plasma membrane can be observed most prominently in the large cells of the vulva and the sheath cells enclosing the spermatheca (Figure 3M, N). In the spermatheca, TSP-17::GFP expression is also clearly enriched around the nucleus (Figure 3N, arrowheads), possibly localizing to the nuclear membrane or endoplasmic reticulum (Figure 3N, arrowhead). Analysis of subcellular localization in the vulva and spermatheca revealed that the TSP-17::GFP *(gt1681)* mutant protein is uniformly expressed in the cytoplasm, with a loss of enrichment at the plasma membrane and around the nucleus (Figure S3A). Thus, the *gt1681* mutation, which leads to an amino acid change in the fourth transmembrane domain, might compromise the membrane localization of TSP-17 and therefore block its function. TSP-17::GFP is also expressed in multiple neurons throughout worm development. For instance, the NSM serotonergic neuron, which is characterized by extensive axon sprouting, shows TSP-17::GFP expression along its entire length (Figure 3O). Prominent expression was also observed in the muscles of early stage larvae (Figure 3P). Finally expression also appears to be apparent in muscles of the adult head (Figure 3B, C, H, I). In summary, the TSP-17::GFP expression indicates that TSP-17 is expressed in dopaminergic neurons. Transgene expression in dopaminergic neurons was also confirmed by analyzing a TSP-17::GFP expressing transgenic strain crossed to a DAT-1 reporter strain (Figure S3B). We cannot rule out expression of TSP-17 not uncovered by the transgene, due to missing regulatory sequences. We next wanted to investigate whether TSP-17 expression in dopaminergic neurons protects them from 6-OHDA-mediated neurodegeneration. By direct injection of transgenes into the gonad, we generated transgenic worms overexpressing TSP-17 under the control of the *dat-1* promoter. Consistent with TSP-17 expression in dopaminergic neurons, we found partial rescue of the hypersensitivity conferred by *gt1681* (Figure 2C, compare bars 1, 2 and 4). Interestingly, overexpression of TSP-17 and TSP-17 (*gt1681*) under the *dat-1* promoter led to spontaneous neurodegeneration (Figure S4A, B, respectively). This phenotype tended to be more severe following TSP-17 *(gt1681)* overexpression. Taken together, these data indicate that TSP-17 indeed functions in dopaminergic neurons, and that excessive TSP-17, especially the mutant form, leads to spontaneous neurodegeneration.

**Figure 4:**
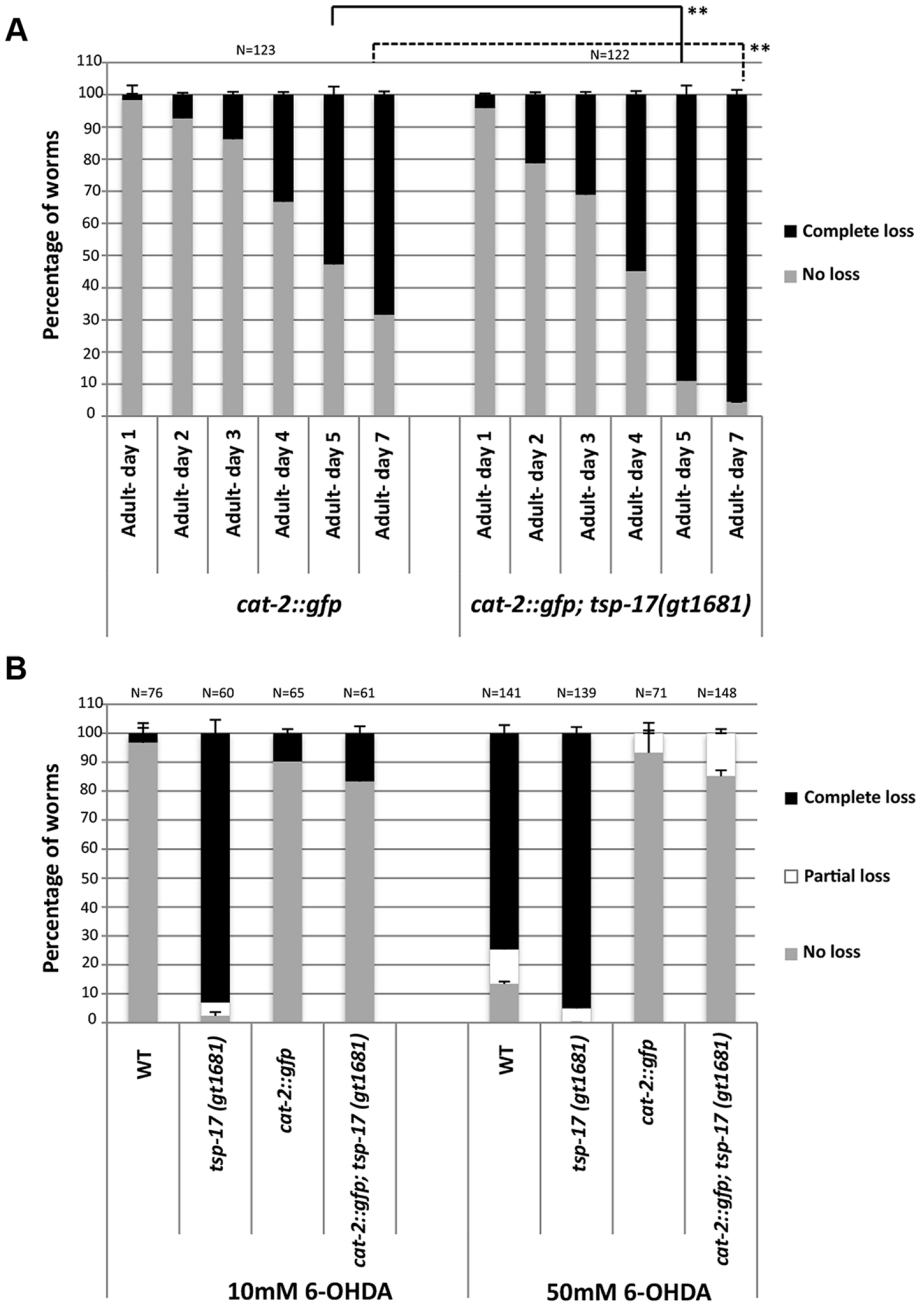
*tsp-17 (gt1681)* enhances the neurodegeneration phenotype of *cat-2*-overexpressing lines, and *cat-2* overexpression protects against 6-OHDA toxicity. **A**. *cat-2* induced neurodegeneration. Analysis of *cat-2*-overexpressing stains UA57 *baIn4 [pdat-1::gfp pdat-1::cat-2]* and TG2402 *baIn4[pdat-1::gfp pdat-1::cat-2]; tsp-17(gt1681)*. Error bars represent standard deviation. Asterisks represent statistical difference between *cat-2::gfp* adults on days 5 and 7 (**p<0.005). **B**. *cat-2* overexpression suppresses 6-OHDA-induced neurotoxicity. Experiments were done in triplicate and the average is shown. Data presented is from scoring the extent of neurodegeneration 72 h post 6-OHDA intoxication.

We next wished to address how TSP-17 protects dopaminergic neurons. We hypothesized that TSP-17 might affect dopamine synthesis, or dopamine and 6-OHDA uptake or degradation. Dopamine metabolism is itself a source of oxidative stress and may initiate ROS-mediated injury to dopaminergic neurons. The link between excessive dopamine exposure and toxicity is controversial, but overexpression of CAT-2, the rate-limiting enzyme in dopamine synthesis in *C. elegans*, is reported to lead to age-dependent degeneration of dopaminergic neurons [46]. We repeated these experiments, and indeed found that neurodegeneration conferred by CAT-2 overexpression in dopaminergic neurons is enhanced in the *gt1681* mutant background (Figure 4A). In contrast, we found CAT-2 overexpression to confer a strong resistance toward 6-OHDA-dependent neurodegeneration in both wild-type and *gt1618* backgrounds (Figure 4B). We consider it likely that 6-OHDA resistance conferred by CAT-2 overexpression can be explained by reduced 6-OHDA uptake into dopaminergic neurons in the presence of excessive levels of intracellular dopamine. Our results indicate that *tsp-17* protects against 6-OHDA toxicity and toxicity caused by excessive dopamine.

**Figure 5:**
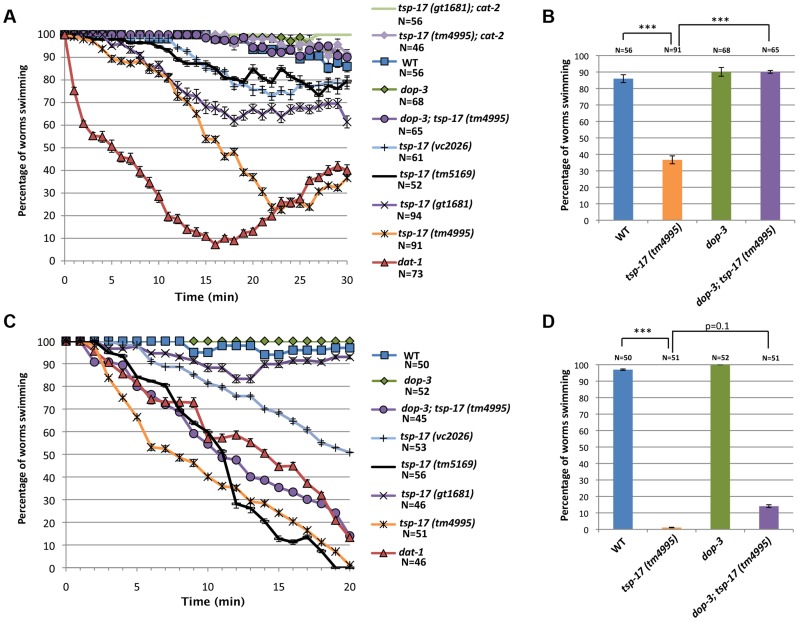
Behavioral phenotypes associated with *tsp-17* mutants. **A**. Quantitative analysis of SWIP behavior at L4-stage, over 30 minutes. **B**. The SWIP phenotype of *tsp-17(tm4995)* in L4-stage worms is rescued by *dop-3* deletion. **C**. Quantitative analysis of SWIP behavior in L1-stage worms over 20 min. **D**. The SWIP phenotype of *tsp-17(tm4995)* at L1 stage is not rescued by *dop-3* deletion. Assays were done in triplicate for the total number of worms indicated by N values. Error bars represent the standard error of the mean. Asterisks represent statistically significant differences from the wild-type (***p<0.01). To facilitate comparison, strains are indicated by the same color code.

Since these genetic interactions suggest that dopamine levels could be altered in *tsp-17* mutants, we next investigated behavioral phenotypes associated with dopamine. Dopamine synthesis and release are required for the basal slowing response, in which worms reduce their speed when encountering a bacterial lawn [36]. We did not observe a defect in this response, indicating that both dopamine synthesis and extracellular dopamine sensing by receptors are intact in *tsp-17* mutants (Figure S5A). One of the most accessible phenotypes thought to be associated with excessive extracellular dopamine is the SWIP (Swimming Induced Paralysis) phenotype [37]. While wild-type worms placed into a drop of water maintain their thrashing frequency *dat-1* mutants become progressively paralyzed. The SWIP phenotype is ascribed to excessive extracellular dopamine as a consequence of the reuptake defect in the *dat-1* mutant. Excessive extracellular dopamine triggers paralysis by hyperactivating the DOP-3 receptor expressed on cholinergic neurons and hence blocking acetylcholine release [33]. To perform this experiment, we placed L4 worms into drops of water and scored their ability to swim over a period of 30 minutes. As expected, we found that wild-type but not *dat-1* mutant worms can swim for 30 minutes with no change in the speed or pattern of swimming. All four *tsp-17* mutants showed a partial SWIP phenotype (Figure 5A). This phenotype is probably caused by dopaminergic signaling because it can be rescued by deletion of the *dop-3* dopamine receptor and by deletion of the *cat-2* tyrosine hydroxylase (Figure 5A and Figure 5B). It was surprising to find a SWIP phenotype in *tsp-17* mutants as we argue that *tsp-17* inhibits *dat-1* function (see below). While elucidating the exact mechanism of how TSP-17 affects behavioral phenotypes will require further investigation we speculate that hyper-activation of DAT-1 in *tsp-17* strains could trigger a feedback loop that transiently enhance extracellular dopamine levels inducing the weak SWIP phenotype we observe.

We also tested for a SWIP phenotype in L1 stage worms, and found that all *tsp-17* mutants tested, except the *gt1681* allele, behaved similarly to *dat-1* mutants (Figure 5C, D). This phenotype, however, is not suppressed by a *dop-3* mutation or blocked by a *cat-2* mutation (Figure 5D and Figure S5B). We discovered that the “L1 SWIP phenotype” is linked to lethality because worms placed onto agar plates after SWIP assay show reduced viability (Figure S5C, D). Thus, the L1 “swimming-induced lethality” phenotype is unlikely to be related to dopamine levels. Given that TSP-17 is expressed in body wall muscles in L1 larvae, we speculate that swimming-induced lethality might be caused by a muscle defect.

**Figure 6:**
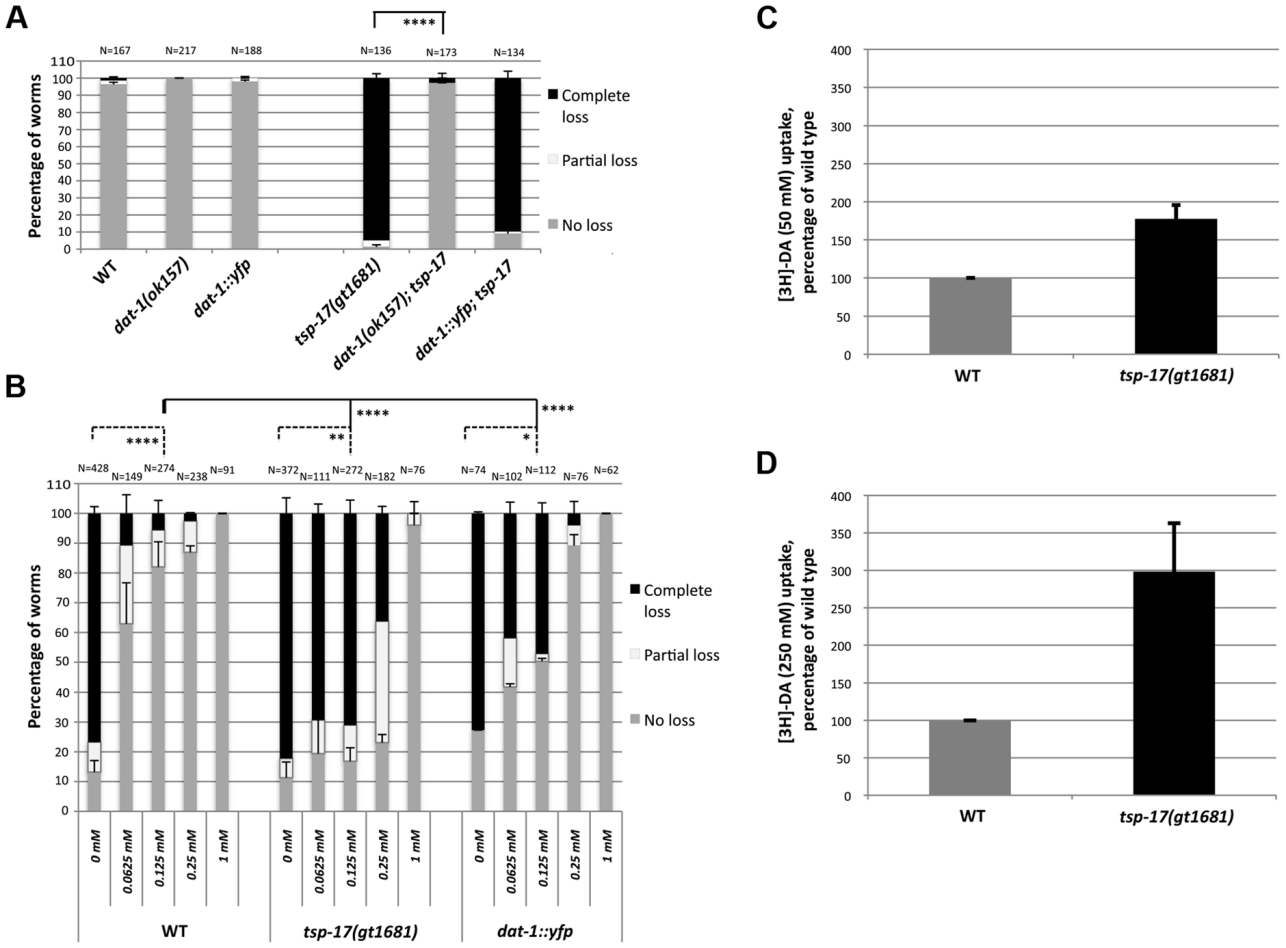
Evidence for DAT-1 hyperactivation in *tsp-17* worms. **A**. *dat-1::yfp* transgenic worms (TG2470) do not exhibit hypersensitivity to 10 mM 6-OHDA. The extent of neurodegeneration was scored 72 h post 6-OHDA intoxication. Asterisks represent statistically significant differences compared to *tsp-17* worms (****p<0.00001) **B**. More imipramine than in wild-type worms is needed to prevent neurodegeneration in *tsp-17* mutants and in *dat-1::yfp* overexpression worms co-treated with 50 mM 6-OHDA. Data presented is from scoring the extent of neurodegeneration 72 h post 6-OHDA intoxication. The imipramine concentration is indicated on the x axis. N, total number of worms from each strain examined for every treatment. Error bars represent the standard error of the mean. Asterisks (below top bar) represent statistically significant differences compared to wild-type; worms treated with 0.125 mM imipramine are compared (****p<0.00001). Lower bars indicate difference within individual strains (no imipramine compared to 0.125 mM imipramine; *p<0.05, *p<0.005, ****p<0.00001) **C., D**. [^3^H]-dopamine (DA) uptake in wild-type and *tsp-17* worms. Uptake assays were performed using 50 nM (**C**) and 250 nM [^3^H]-DA (**D**).

To systematically test whether TSP-17 protects dopaminergic neurons by modulating dopamine metabolism, catabolism, reuptake or signaling, we performed a genetic epistasis analysis. As expected, *tsp-17 dat-1* double mutants were completely resistant to 6-OHDA-induced neurodegeneration, consistent with the notion that TSP-17 does not bypass 6-OHDA uptake by the DAT-1 dopamine transporter (Figure 6A). We observed no alterations in 6-OHDA sensitivity in *cat-2* (tyrosine hydroxylase), *bas-1* (aromatic amino acid decarboxylase/AAADC) and *cat-1* (VMAT ortholog required for dopamine packaging) *tsp-17* double mutants, indicating that TSP-17 is unlikely to affect levels of dopamine synthesis or packaging (Figure S6).

As 6-OHDA can enter dopaminergic neurons through the DAT-1 transporter owing to its structural similarity to dopamine [3], [47], we wondered whether DAT-1 localization or activity is modified in a *tsp-17* mutant background. Having established that 6-OHDA hypersensitivity in *tsp-17* worms depends on the DAT-1 transporter (Figure 6A), we tested the hypothesis that enhanced DAT-1 transporter activity may contribute to enhanced 6-OHDA-mediated neurotoxicity. Using a functional p*dat-1*::*dat-1::YFP* translational fusion, we found that overexpression of this transgene generated by bombardment does not confer overt 6-OHDA hypersensitivity (Figure 6A, Figure S7). Furthermore, the localization of DAT-1::YFP was similar between wild-type and *tsp-17* mutants worms (Figure S8A), a notion further confirmed by Structural Illumination ‘super resolution’ images of CEP dendrites (Figure S8B). Additionally, photobleaching experiments indicated that ∼half of DAT-1::YFP is in the mobile fraction and that the t1/2 is around 30 seconds in both wild-type and *tsp-17(gt1681)* worms (Figure S8C–E). We thus aimed to test whether TSP-17 negatively regulates DAT-1 activity using a pharmacological approach. We confirmed previous reports that imipramine specifically inhibits the DAT-1 transporter in the worm [3] (Figure 6B, left panels, wild-type 0.25 mM and 1 mM). We reasoned that if DAT-1 is hyperactive in *tsp-17* (*gt1681*), relatively more imipramine should be needed to inhibit DAT-1 activity and prevent neurodegeneration. We thus treated wild-type, *tsp-17 (gt1681) worms* and wild-type worms overexpressing *DAT-1::YFP* with 10 mM 6-OHDA and increasing doses of imipramine (Figure 6B, middle and right panels). We indeed found that higher levels of imipramine are needed to reduce neurodegeneration in DAT-1::YFP overexpressing worms and in *tsp-17 (gt1681)* worms, and that the effect being stronger in the *tsp-17 (gt1681)* mutant. Reduced levels of neurodegeneration levels were most clearly observed when concentrations of 0.125 mM and 0.25 mM imipramine were used (Figure 6B). This result provides evidence that DAT-1 activity may be higher in the *tsp-17* mutant background. We aimed to provide further support for this hypothesis by directly measuring dopamine uptake, following previously described procedures. We macerated *C. elegans* embryos to establish primary embryonic cell cultures, and used these for dopamine uptake assays [48], [49]. Using two concentrations of tritiated dopamine, we indeed found increased dopamine uptake in *tsp-17* mutants (Figure 6C, D). We note that we found this in 7/8 repeat experiments. However, we also note that only a very small proportion of tissue culture cells are dopaminergic neurons and that the absolute amount of dopamine uptake is low especially in the wild-type background.

**Figure 7:**
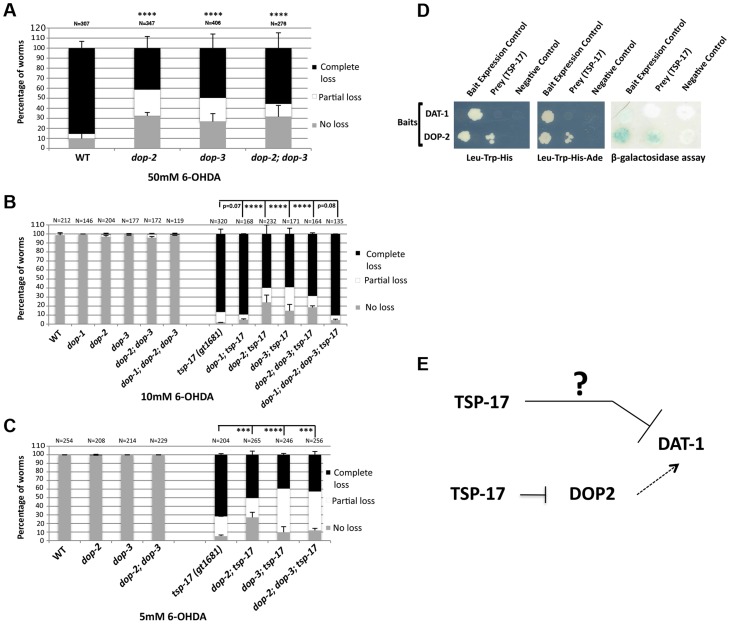
Dopamine receptors act antagonistically to modulate the sensitivity of *tsp-17 (gt1681)* mutants to 6-OHDA. Worms of the indicated genotypes were intoxicated with **A**. 50 mM, **B**. 10 mM 6-OHDA and **C**. 5 mM 6-OHDA and scored for neurodegeneration 72 h post intoxication. Experiments were done in triplicate and the average data is presented. N, total number of animals examined for each strain. Error bars represent the standard error of the mean. Asterisks represent statistically significant differences (***p<0.0001, ****p<0.00001). **D**. Evidence for a direct interaction between DOP-2 and TSP-17. Growth on -Leu, -Trp, -His (left panel) and -Leu, -Trp, -His, -Ade (middle panel) plates is shown. The right panel depicts a β-galactosidase assay. **E**. Working model as to how TSP-17 might interact with DAT-1 and DOP-2 to modulate level of DAT-1 activity. Arrows indicate activation. T-bars indicate repression. The question mark indicates that we do not know the mechanism of DAT-1 inhibition by TSP-17.

Our combined genetic, pharmacological and biochemical analysis suggests that TSP-17 modulates DAT-1 activity. Previous studies using tissue culture-based assays demonstrated that dopamine receptor activation might promote DAT-1 activity [25], [50], [51]. Consistent with these results, we found *dop-2* and *dop-3* mutant worms to be partially resistant to high doses of 6-OHDA compared to wild-type (Figure 7A). We therefore investigated whether *tsp-17* genetically interacts with dopamine receptors to modify DAT-1 activity and confer differential 6-OHDA sensitivity. This was done by assessing the sensitivity of *tsp-17* mutants in the absence of the *C. elegans* DOP-1 D1-like receptor and/or in the absence of the DOP-2 and/or DOP-3 D2-like receptors. *C. elegans* DOP-1 is expressed in a variety of cells, including cholinergic neurons, mechanosensory neurons, head muscles and neuronal support cells. DOP-3 is expressed postsynaptically and its antagonism of DOP-1 in cholinergic neurons is required for the regulation of locomotion [33]. The DOP-2 receptor is expressed both postsynaptically and presynaptically. When expressed presynaptically, it acts as an autoreceptor on the plasma membrane of dopaminergic neurons. We found that *dop-1*; *tsp-17 (gt1681)* was as sensitive to 6-OHDA as the respective *tsp-17* single mutant. In contrast, 6-OHDA hypersensitivity was reduced in *dop-2*; *tsp-17 (gt1681)* and *dop-2*; *tsp-17 (tm4994)* and in *dop-3*; *tsp-17 (gt1681)* and *dop-3; tsp-17 (tm4994)* double mutant worms (Figure 7B, C and Figure S9) Our genetic data thus argue that TSP-17 might inhibit DOP-2 and DOP-3 function, which in turn might be required for full DAT-1 transporter activity (Figure 7A, E). Given that deletion of *dop-2* and *dop-3* only partially rescues 6-OHDA hypersensitivity in *tsp-17* mutants, we speculate that TSP-17 also inhibits DAT-1 activity independently of DOP-2 and DOP-3.

We next aimed to investigate how TSP-17 might regulate DAT-1 or D2-like receptors to modulate DAT-1 activity. Given that these are integral membrane proteins, we employed the split-ubiquitin membrane-based yeast two-hybrid system [52]. In this system, a C-terminal ubiquitin moiety fused to a transmembrane protein and a transcription factor is used a bait. An N-terminal ubiquitin moiety is used as the “prey.” Upon “reconstruction” of the split ubiquitin, this molecule is recognized by a protease, which cleaves the transcription factor, thus promoting reporter gene activation. By employing various bait and prey fusions with TSP-17, DAT-1 and DOP-2, we could not find a direct interaction between TSP-17 and DAT-1 using the split-ubiquitin system (Figure 7D). In contrast, we found that DOP-2 and TSP-17 may indeed interact. The specificity of this interaction was clearly revealed when the beta-galactosidase reporter assay was used as an output. In addition, yeast colony formation on his-3 or his-3 ade-2 plates was enhanced when the corresponding reporters where used (Figure 7D). Thus, TSP-17 might modulate DOP-2 activity by a direct physical interaction, consistent with TSP-17 affecting ligand binding, downstream signaling or membrane trafficking of DOP-2-like receptors. Our genetic data also suggest that TSP-17 might also act via other factors to dampen DAT-1 activity (Figure 7B).

## Discussion

Using *C. elegans* as a model and employing unbiased genetic approaches, we aimed to find neuroprotective genes that alleviate the 6-OHDA-induced degeneration of dopaminergic neurons. Based on our genetic data, which is supported by the characterization of several alleles and transgenic rescue experiments, we provide compelling evidence that TSP-17 protects dopaminergic neurons from 6-OHDA-mediated toxicity. TSP-17 appears to function in dopaminergic neurons, and our combined genetic, pharmacological and biochemical evidence suggests that it might act by antagonizing DAT-1 dopamine transporter activity. We do not know how TSP-17 regulates DAT-1 at a mechanistic level.

TSP-17 is a member of the evolutionarily conserved family of tetraspanins, comprising 20–50 kDa membrane proteins that contain four transmembrane domains. A characteristic feature of tetraspanins is their ability to form lateral associations with each other and with other proteins. Such interactions are thought to lead to a dynamic assembly, resulting in the formation of a network of molecular interactions referred to as the tetraspanin web [41], [53]. Tetraspanins are thought to have regulatory functions in the ligand binding, downstream signaling, protein trafficking and proteolytic activities of associated proteins [42], [54]. In *C. elegans*, only two tetraspanins have known functions. TSP-15 appears to be required to activate the BLI-3 dual oxidase to regulate H_2_0_2_ production at the plasma membrane and thus alter dityrosine cross-linkage of extracellular matrix proteins [44], [55]. Genetic evidence suggests that TSP-12, most closely related to human TSPAN33, appears to facilitate Notch signaling redundantly with TSP-14. Thus conserved tetraspanins likely function by facilitating γ-secretase cleavage of the membrane-bound form of Notch, thus promoting nuclear localization of this transcription factor [43].

DAT-1 hyperactivity in the *tsp-17* mutants could result from altered DAT-1 localization or abundance at the cell membrane; alternatively, TSP-17 might indirectly regulate DAT-1 activity. Using a functional DAT-1::YFP construct, we did not see any obvious change in DAT-1 expression, localization, or change in half life in *tsp-17* mutants and we thus favor the idea that TSP-17 regulates DAT-1 activity. Our finding that TSP-17 genetically and biochemically interacts with the DOP-2 D2-like dopamine receptor, suggests an indirect mode of DAT-1 regulation by TSP-17 (Figure 7E). Our genetic analysis provides evidence that TSP-17 might in part regulate DAT-1 via DOP-2 and DOP-3 dopamine receptors (Figure 7E). We found that depletion of the D2-like dopamine receptors, DOP-2 and/or DOP-3, in *tsp-17* mutants leads to a moderate reduction in the 6-OHDA hypersensitivity conferred by *tsp-17*, while D2-like dopamine receptor single knockout strains show the same 6-OHDA sensitivity as wild-type worms. Thus, our analysis suggests that *tsp-17* genetically interacts with D2-like dopamine receptors, in line with our observation that TSP-17 directly binds to DOP-2. In mammalian systems, dopamine autoreceptors are reported to have a major role in providing inhibitory feedback to adjust the rate of neuronal firing, dopamine synthesis and dopamine release in response to the dopamine level in the synaptic cleft [30], [32]. Several studies suggest that vertebrate D2 dopamine receptors also modulate DAT-1 activity to regulate the dopamine level in the synaptic cleft. Cass and Gerhardt used pharmacological approaches to demonstrate that inhibition of D2 class dopamine receptors significantly inhibits DAT function [50]. Two independent studies provided evidence that D2 receptors regulate both the activity and cell surface expression of DAT-1 [25], [51]. Nevertheless, further investigations are required to establish functional links between *C. elegans* DOP-2 receptors and DAT-1 activity. The ability of TSP-17 to inhibit DAT-1 both via DOP-2 and independent of D2-like receptors (Figure 7E) suggests that TSP-17 modulates the activity of multiple signaling proteins. Indeed, our observation of excessive neurodegeneration following wild-type, and especially mutant, TSP-17 overexpression in dopaminergic neurons hints that malfunctioning and/or excessive TSP-17 blocks pathways needed to maintain the integrity of dopaminergic neurons. The enhanced defect associated with overexpression of mutant TSP-17 that fails to show the correct cytoplasmic localization hints the neurotoxicity might be conferred by the sequestration of TSP-17 interacting proteins essential for neuronal survival.

Dopamine neuronal dysfunction has been associated with several common neurobehavioral disorders, including drug addiction, schizophrenia and attention-deficit hyperactivity disorder [32], [56]–[58]. The DAT-1 dopamine transporter plays a central role in dopamine signaling, and it is likely to be subjected to complex modes of regulation. DAT-1 is the target of psychoactive addictive drugs such as cocaine and amphetamine, and DAT1 overexpression leads to increased amphetamine sensitivity [59]–[63]. Mechanisms related to dopamine signaling tend to be evolutionarily conserved. Thus, studies aimed to genetically define modulators of dopamine signaling and 6-OHDA-mediated toxicity will provide important insights into the mechanisms of dopamine signaling in health and disease.

Idiopathic PD is thought to be triggered by a combination of environmental factors and genetic susceptibility, and a case has been made that exposure to environmental toxins such as the pesticides paraquat and rotenone leads to increased PD [9]. Indeed, chemical and tissue culture studies have provided evidence that increased dopamine levels may lead to enhanced neurodegeneration, probably through the generation of toxic intermediates such as the neurotoxic product of dopamine oxidation, 6-OHDA [13], [15], [64]–[68]. The specificity of 6-OHDA entry into dopamine neurons depends on DAT, and DAT antagonists can block uptake [3], [4], [11], [47]. Interestingly, DAT-1 hyperactivity in *tsp-17* mutants further enhances the neurodegeneration conferred by elevated dopamine synthesis in CAT2 tyrosine hydroxylase-overexpressing worm strains. Thus, DAT-1 hyperactivity might enhance neurodegeneration by further increasing the intracellular concentration of dopamine and/or toxic metabolites. DAT1 expression or activity has not been linked to PD, but it is intriguing that among dopamine neurons those residing in the substantia nigra express the highest DAT levels in vivo and are most strongly affected in PD [4], [60].

## Materials and Methods

### *C. elegans* strains and maintenance

Strains were grown at 20°C under standard conditions, unless indicated otherwise. N2 Bristol was used as the wild-type strain. The *tsp-17*(*tm4994*) and *tsp-17*(*tm5169*) mutants were generated and kindly provided by Shohei Mitani of the National Bioresource Project for the Nematode (http://www.shigen.nig.ac.jp/c.elegans/). Details of the respective alleles are described by the National Bioresource Project for the Nematode and by WormBase (www.wormbase.org). All mutants were outcrossed a minimum of four times to the TG2435 *vtIs1*[p*dat-1::gfp*] strain originally generated by the Blakely laboratory (BY200) and repeatedly crossed into the N2 background.

### Strains

**TG2435** *vtIs1[pdat-1::gfp; rol-6]* V,

**TG1681** *vtIs1* V; *tsp-17(gt1681)* X,

**TG2436** *vtIs1* V; *tsp-17(tm4994)* X,

**TG2437** *vtIs1* V; *tsp-17(tm5169)* X,

**TG2438** *vtIs1* V; *tsp-17(gk276386)* X,

**TG2462** *vtIs1* V; CB4856,

**TG2463** *vtIs1* V; *lon-2(e678) unc-20(e112) X,*

**TG2464** *vtIs1* V; *tsp-17(gt1681) unc-20(e112) X,*

**TG2465** *vtIs1* V; *tsp-17(gt1681) lon-2(e678) X,*

**TG2395** *cat-2(e1112)* II; *vtIs1* V,

**TG2394** *cat-2(e1112)* II; *vtIs1* V; *tsp-17(gt1681)* X,

**TG2396** *bas-1(tm351)* III; *vtIs1* V,

**TG2397** *bas-1(tm351)* III; *vtIs1* V; *tsp-17(gt1681)* X,

**TG2399** *vtIs1* V; *cat-1(e1111)* X,

**TG2398** *vtIs1* V; *cat-1(e1111) tsp-17(gt1681)* X,

**TG2400** *dat-1(ok157)* III; *vtIs1* V,

**TG2401** *dat-1(ok157)* III; *vtIs1* V; *tsp-17(gt1681)* X,

**TG2404** *amx-1(ok659)* III; *vtIs1* V,

**TG2403** *amx-1(ok659)* III; *vtIs1* V; *tsp-17(gt1681)* X,

**TG2406** *amx-2(ok1235)* I; *vtIs1* V,

**TG2405** *amx-2(ok1235)* I; *vtIs1* V; *tsp-17(gt1681)* X,

**TG2408** *amx-2(ok1235)* I; *amx-1(ok659)* III; *vtIs1* V,

**TG2407** *amx-2(ok1235)* I; *amx-1(ok659)* III; *vtIs1* V; *tsp-17(gt1681)* X,

**TG2410** *vtIs1* V; *dop-1(vs100)* X,

**TG2409** *vtIs1* V; *dop-1(vs100) tsp-17(gt1681)* X,

**TG2412** *vtIs1 dop-2(vs105)* V,

**TG2411** *vtIs1 dop-2(vs105)* V; *tsp-17(gt1681)* X,

**TG2414** *vtIs1* V; *dop-3(vs106)* X,

**TG2413** *vtIs1* V; *dop-3(vs106) tsp-17(gt1681)* X,

**TG2466** *vtIs1 dop-2(vs105)* V; *dop-3(vs106)* X,

**TG2467** *vtIs1 dop-2(vs105)* V; *dop-3(vs106) tsp-17(gt1681)* X,

**TG2415** *vtIs1 dop-2(vs105)* V; *dop-1(vs100) dop-3(vs106)* X,

**TG2416** *vtIs1 dop-2(vs105)* V; *dop-1(vs100) dop-3(vs106) tsp-17(gt1681)* X,

**UA57** *baIn4[pdat-1::gfp pdat-1::cat-2],*

**TG2402** *baIn4[pdat-1::gfp pdat-1::cat-2],; tsp-17 (gt1681)* X,

**TG2470** *gtIn2469*[*pdat-1*::*dat-1::yfp*::*let-858* 3′UTR, *unc-119*(+)]; *gtIn2468*[*pdat-1:*:*mcherry*::*let858* 3′UTR, *unc-119*(+)]; *unc-119(ed3)* III,

**TG2471** *gtIn2469*[*pdat-1*::*dat-1::yfp*::*let-858* 3′UTR, *unc-119*(+)]; *gtIn2468*[*pdat-1:*:*mcherry*::*let858* 3′UTR, *unc-119*(+)]; *unc-119(ed3)* III; *tsp-17(gt1681) X*,

**TG2439** *gtIn2439*[*ptsp-17:*:*tsp-*17::*gfp*::*tsp-17* 3′UTR, *pdat-1::mcherry:*:*let858* 3′UTR, *unc-119*(+)]; *unc-119(ed3)* III,

**TG2472** *tsp-17(gt1681)* X; *gtIn2439*[*ptsp-17:*:*tsp-*17::*gfp*::*tsp-17* 3′UTR, *pdat-1::mcherry:*:*let858* 3′UTR, *unc-119*(+)]; *unc-119(ed3)* III,

**TG2440** *gtEx2440[pdat-1::tsp-17::cfp:: let-858* 3′UTR, *unc-119(+)]; unc-119(ed3)* III; *vtIs1* [*pdat-1::gfp; rol-6*] V,

**TG2473** *vtIs1* [*pdat-1::gfp; rol-6*] V; *tsp-17(gt1681)* X; *gtEx2440 [pdat-1::tsp-17::cfp:: let-858* 3′ UTR, *unc-119(+)],*

**TG2474** *vtIs1* [*pdat-1::gfp; rol-6*] V; *unc-119(ed3)* III; *gtEx2474[pdat-1::tsp-17(*G74E*)::cfp:: let-858* 3′UTR, *unc-119(+)],*

**TG2478** *cat-2(e1112)* II; *vtIs1*V; *tsp-17(tm4994)* X,

**TG2475** *dat-1(ok157)* III; *vtIs1*V; *tsp-17(tm4995)* X,

**TG2477** *vtIs1*; *dop-3(vs106) tsp-17(tm4995)* X,

**TG2476** *dat-1(ok157)* III; *vtIs1*V; *dop-3(vs106)* X,

#### Generation of transgenic worms and constructs

NM001, NM002, Pb1001, PbI002, PbI003 and AH001 plasmid sequences can be obtained upon request.

Plasmids generated in this study are as follows:

**NM001** pRH21-*ptsp-17*::*tsp-17::gfp*::*tsp-17* 3′UTR

**NM002** pRH21-*ptsp-17::tsp-17(gt1681)::gfp*::*tsp-17* 3′UTR

**PbI001** pRH21-*pdat-1*::*mcherry*::*let858* 3′UTR

**PbI002** pRH21-*pdat-1*::*tsp-17::cfp*::*tsp-17* 3′UTR

**PbI003** pRH21-*dat-1*::*tsp-17(gt1681)::cfp:*:*tsp-17* 3′UTR

**AH001** pRH21-p*dat-1*::*dat-1::yfp*::*let-858* 3′UTR

**NM003** pBT3-STE-*dop-2c*-Cub

**NM004** pBT3-STE-*dat-1*-Cub

**NM005** pPR3-STE-*tsp-17*1b-NubG

Plasmids were generated using the following primers:

*dat-1*_pmt_**AscI**_F, atatGGCGCGCCaatgtttctagtcgtttttgta

*dat-1*_pmt_**SgfI**_R, ctccGCGATCGCggctaaaaattgttgagattcg

*mCherry_* **NotI**_F, ggagGCGGCCGCatggtctcaaagggtgaagaag

*mCherry_* **FseI**_R, cctaGGCCGGCCccttatacaattcatccatgccacc

F_pmt-*tsp-17*_**AscI**, agtcGGCGCGCCagtctgaaaaacaacagagttagatg

F_ATG_**SgfI_** *tsp-17a*_Cter, ggagGCGATCGCatgcttctcgacccgaaac

R_*tsp-17gnc*_NO-TAA_**cNotI**, atgcGCGGCCGCcgtagtcatctcgaattacatgg

F_**PacI**_TAA-3utr_*tsp17*, gtacTTAATTAAtaaatcactctacggtgaatta

R_**ApaI**_3utr_*tsp-17*, cagtGGGCCCtcactaatatatgttctcagtcc

GFP-CFP_**NotI**_F, GCGGCCGCatgagtaaaggagaagaacttttc

GFP_**FseI**_R, GGCCGGCCccttgtatggccggctagcg

F_dop-2c_pBT3STE, gctaGGCCATTACGGCCgaggccggagagacatggaat

R_dop2c_pBT3STE, gctaGGCCGAGGCGGCCccgacatgcgcctgcttgttact

F_dat-1_pBT3STE, gctaGGCCATTACGGCCCAGTTGGTGCCTACAGACGAT

R_dat-1_pBT3STE, CCGCACTCTGACATAATGCTAggGGCCGCCTCGGCCtagc

F_tsp-17_pPR3STE, gctaGGCCATTACGGCCTTgcaacagaacgtgatggc

R_tsp-17_pPR3STE, gtcaGGCCGAGGCGGCCCCgtagtcatctcgaattacatggta

The TG2470 and TG2439 strains were generated by biolistic bombardment of *unc-119*(*ed3*) worms with AH001 and NM001 plasmids, respectively. The TG2440 and TG2474 strains were generated by microinjections of *unc-119 (ed3)* mutants.

### Mutagenesis and mapping

EMS was added to 4 ml synchronized young adult worms in M9 buffer to a final concentration of 25 mM and incubated for 4 h at 20°C. Mutagenized worms were washed in M9 buffer and incubated at 15°C. Synchronous F1-generation L1 larvae were used for screening. F2-generation L1 larvae from mutagenized TG2435 *dat-1::gfp* (*BY200*) worms were used for the mutagenesis screen. L1 larvae were intoxicated with 10 mM 6-OHDA. After 72 h, worms with the highest incidence of neurodegeneration were isolated and scored as hypersensitive. SNP mapping of mutants was done as previously described [69].

### Drug treatment of worms

To obtain synchronized L1 larvae, 1–10 adult worms (24 h post-L4 stage) were incubated in 70 µl M9 without food on at 20°C, with shaking at 500 rpm for 27–40 h to lay eggs. After hatching, all L1 larvae were collected. Approximately 50 L1 larvae were added to an assay mix (50 µl) containing 10 mM 6-OHDA and 40 mM ascorbic acid, and incubated for 1 h at 20°C, with shaking at 500 rpm. For co-treatment with imipramine or haloperidol, the respective compounds were added to the assay mix at the same time as 6-OHDA. After a 1-h incubation, M9 buffer (100 µl) was added to the assay mix, and the solution containing L1 worms was then transferred to an unseeded NGM plate. After 30 min, L1 worms were individually picked and transferred onto a fresh NGM plate seeded with a line of OP50 bacteria to ease subsequent scoring. Intoxicated worms were incubated at 20°C and scored for dopaminergic neurodegeneration every 24 h for 3 days. All 6-OHDA treatments were done in triplicate and at least 80–100 worms were tested for each strain and condition.

### Swimming-induced paralysis assay

All worms used for SWIP analysis were grown on NGM plates seeded with *E. coli* OP50 bacteria. For each test, 5–10 L4 hermaphrodites or 10 L1 worms were placed into 40 µl water in a single well of a Pyrex Spot Plate. Paralyzed worms were counted at 1-min intervals using a Leica dissecting microscope [70]. L1 worms were hand picked from seeded plates, 12 hours after the addition of embryos, obtained by bleaching.

### Scoring neuronal degeneration and image acquisition

For semi-quantitative analyses of 6-OHDA-induced degeneration, worms were examined using a Leica fluorescent dissecting microscope. The absence of all eight dopaminergic neurons in worms was scored as “complete loss.” The presence of a complete, intact set of eight dopaminergic neurons was scored as “no loss.” Any intermediate situation, for example a damaged or absent subset of dopaminergic neurons or missing dendrite portions, was scored as a “partial loss.” Neurodegeneration resulting from *cat-2* overexpression was scored using developmentally synchronized worms, as indicated. A DeltaVision microscope (Applied Precision) was used to acquire images. All images were analyzed using softWoRx Suite and softWoRx Explorer software (Applied Precision).

### DNA constructs for the split-ubiquitin system

Total RNA was isolated and reverse transcribed from wild-type *C. elegans* (N2) using an RNeasy mini kit (QIAGEN). Coding regions of *dop-2c* (K09G1.4c) and *dat-1* (T23G5.5) were amplified and cloned into pBT3-STE vectors (Dual Systems Schlieren) for expression of a fusion protein containing the C-terminal half of ubiquitin (Cub) and the artificial transcription factor LexA-VP16. *tsp-17b* (C02F12.1b) cDNA was amplified and cloned into prey vector pPR3-STE for expression of a fusion protein containing a mutated version of the N-terminal half of ubiquitin (NubG). Constructs were verified by DNA sequencing, and sequences of the respective constructs can be provided upon request. Yeast transformations and pairwise interaction assays were done according to the protocol of Dualsystems Schlieren.

### *C. elegans* cell culture and DAT-1 uptake assay

Embryonic cells were prepared as described previously (Christensen, M, et al 2002, Neuron). The uptake assay was done according to Carvelli et al. (2004). Briefly, *C. elegans* cells cultured for 2 days were washed twice with KRH buffer (120 mM NaCl, 4.7 mM KCl, 1.2 mM KH2P04, 10 mM Hepes, 2.2 CaC12, 10 mM glucose, 0.1 mM ascorbic acid and 0.1 mM tropolone and 0.1 mM pargyline mono amine oxidase inhibitors) and incubated with 50 or 250 nM [^3^H]-dopamine for 20 min at room temperature. Uptake was terminated by three washes of ice-cold KRH buffer, and cells were lysed by incubation with 1% SDS for 20 min. [_3_H]-dopamine uptake was measured in each genetic background, based on radioactive counts, using a scintillation counter (PerkinElmer Liquid Scintillation Analyzer Tri-Carb 1800TR). Total cell numbers were determined with a hemocytometer and were used to normalize radioactive counts. Cell numbers varied between experiments but were not biased towards mutant or control strain: There were 400,000/400,000, 75,000/150,000 and 1,000,000/400,000 cells for control/mutant strain, respectively. Cell extraction and uptake assays were always done simultaneously for both strains. The error bars depict the standard error of the means (SEM).

### Statistical analysis

Neurodegeneration and SWIP assay data are presented as the average of three biological replicates, and error bars represent the standard error of the mean, unless otherwise indicated. When assaying neurodegeneration statistical significance was calculated using the Chi-Sqare test using Yates p-values. http://www.quantpsy.org/chisq/chisq.htm. The statistical significance of differences in the SWIP assays (Figure 5) was calculated using the two-tailed t-test.

## Supporting Information

Figure S1 **Neurodegeneration induced by various doses of 6-OHDA, scored 72 h post intoxication.**
(TIF)

Figure S2 **Phylogenetic analysis of TSP-17.** For phylogenetic analysis, sequences were aligned by ClustalW using Jalview software and an un-rooted phylogenetic tree was generated using SplitsTree. Bootstrap values at the center of the tree (magnified in the red box) indicate divergence. Abbreviations are as follows. As, *Ascaris suum*; Hm, *Hydra magnipapillata*; Ix, *Ixodes scapularis*; Pp, *Pristionchus pacificus*; Hs, *Homo sapiens*; Nv, *Nematostella vectensis*; Dm, *Drosophila melanogaster*; Bm, *Brugia malai*; Ci, *Ciona intestinalis*; Ce, *Caenorhabditis elegans*; Cbn, *Caenorhabditis brenneri*; Cre, *Caenorhabditis remanei*; Cbr, *Caenorhabditis briggsae*. *C. elegans* TSP-17 is highlighted by a red box.
(TIF)

Figure S3 **A. TSP-17(GT1681)::GFP expression in the vulva (left panel) and the spermatheca (right panel).** Strain TG2474 was used. Images are projections of six Z-stacks. **B. TSP-17 expression in ADE and CEP cell bodies**.
(TIF)

Figure S4 **TSP-17 overexpression in a wild-type background induces neurodegeneration without 6-OHDA treatment.** Strains used were (**A**) TG2440 for TSP-17 overexpression and (**B**) TG2474 TSP-17(gt1681) overexpression.
(TIF)

Figure S5 **Analysis of** ***tsp-17*** **behavioral phenotypes. A. Basal slowing response. Movement before (grey bars) and after reaching a lawn of bacteria (white bars) is indicated. B. Quantitative analysis of SWIP behavior in L1-stage worms, over 20 min.** The SWIP phenotype of L1-stage *tsp-17(tm4995)* worms is not rescued by *cat-2*. Assays were done in triplicate. Error bars represent the standard error of the mean. **C, D**. **L1 “swimming-induced lethality” phenotypes.** Worms were incubated as for the L4 swimming induced paralysis assay and plated on seeded plates after the indicated times to assess viability. Representative pictures are shown in D.
(TIF)

Figure S6 **Analysis of 6-OHDA mediated neurodegeneration in** ***cat-2*** **,** ***bas-1*** **and** ***cat-1*** **strains.** Data presented is from scoring the extent of neurodegeneration 72 h post 6-OHDA intoxication.
(TIF)

Figure S7 **Dopamine receptors act antagonistically to modulate the 6-OHDA sensitivity of** ***tsp-17(tm4995)*** **mutants.** Worms of the indicated genotypes were intoxicated with the indicated doses of 6-OHDA and scored 72 h after intoxication. Experiments were done in triplicate and the average data is presented.
(TIF)

Figure S8 **DAT-1::YFP expression and half live is not altered in** ***tsp-17(gt1681)*** **mutant worms.** **A**. Expression of TSP-17 in CEP neurons in wild-type (TG2470) and *tsp-17(gt1681)* mutants (TG2471). **B**. Structural Illumination ‘super resolution’ images of a CEP dendrite in wild-type and *tsp-17(gt1681)* worms showing membrane localization of DAT-1::YFP relative to a cytoplasmic mCherry marker. There are no differences in expression. The crosshatching-like pattern is an artifact introduced by the diffraction grid used in acquisition, not a feature of expression. Scale bar (white) is 5 µm in length. Images are 18 µm×18 µm. **C**. Representative FRAP images of DAT-1::YFP taken prior to bleaching (−6 s), immediately after the bleach event (2 s) and after 2 minutes post bleaching (120 s). Images are 18 µm×18 µm. **D**. Representative graphs showing normalized recovery curves in wild-type (top) and *tsp-17(gt1681)* (bottom) worms. Example half time of recovery (t1/2) for each graph is shown at the intersection of the dashed lines. The mobile fraction is the point at which the curve plateaus. **E**. Average values and standard deviation for t1/2 and mobile fractions for DAT-1::YFP wild-type and *tsp-17(gt1681)* worms (n = 7).
(TIF)

Figure S9 **Dopamine receptors act antagonistically to modulate the 6-OHDA sensitivity of** ***tsp-17(tm4995)*** **mutants.** Worms of the indicated genotypes were intoxicated with 10 mM 6-OHDA and scored 72 h after intoxication. Experiments were done in triplicate and the average data is presented.
(TIF)
