# Supplementary figures and images for: Tetraspanin (TSP-17) Protects Dopaminergic Neurons against 6-OHDA-Induced Neurodegeneration in C. elegans
Source: PLoS Genet. 2014 Dec 4;10(12):e1004767. doi: 10.1371/journal.pgen.1004767 (PMC4256090; doi:10.1371/journal.pgen.1004767)

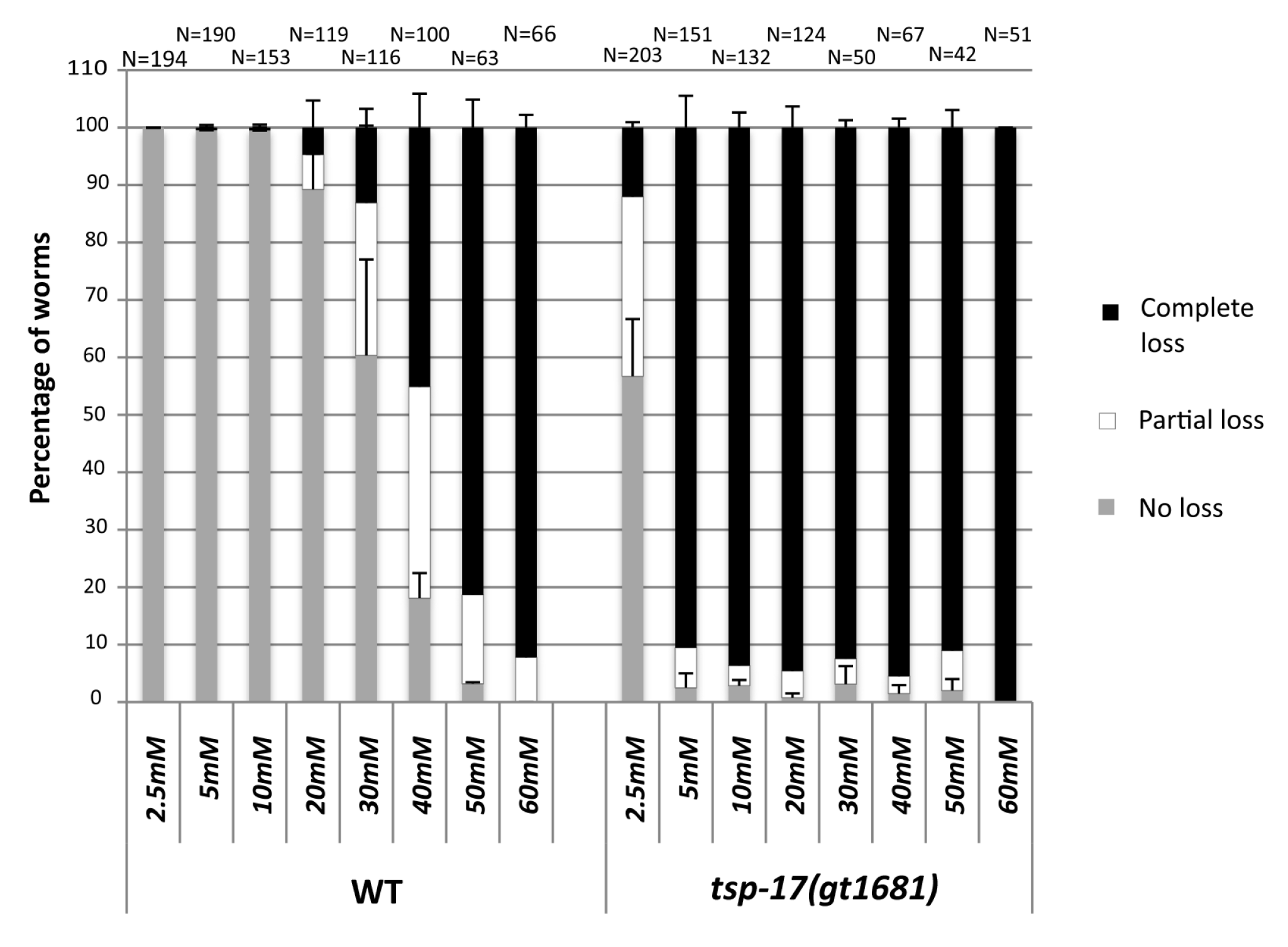

Supplement: Figure S1 — Neurodegeneration induced by various doses of 6-OHDA, scored 72 h post intoxication. (TIF) [file pgen.1004767.s001.tif]

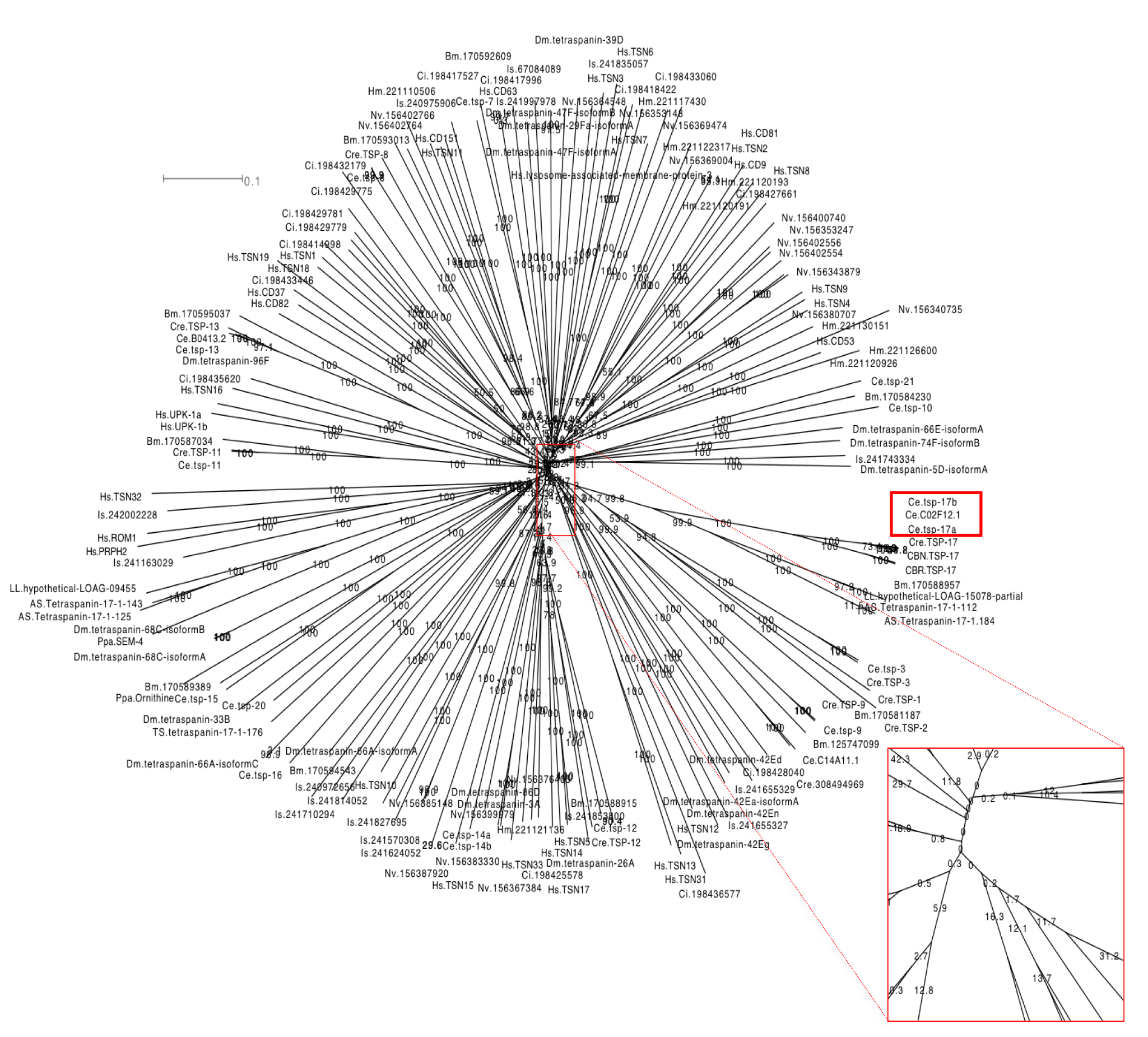

Supplement: Figure S2 — Phylogenetic analysis of TSP-17. For phylogenetic analysis, sequences were aligned by ClustalW using Jalview software and an un-rooted phylogenetic tree was generated using SplitsTree. Bootstrap values at the center of the tree (magnified in the red box) indicate divergence. Abbreviations are as follows. As, Ascaris suum; Hm, Hydra magnipapillata; Ix, Ixodes scapularis; Pp, Pristionchus pacificus; Hs, Homo sapiens; Nv, Nematostella vectensis; Dm, Drosophila melanogaster; Bm, Brugia malai; Ci, Ciona intestinalis; Ce, Caenorhabditis elegans; Cbn, Caenorhabditis brenneri; Cre, Caenorhabditis remanei; Cbr, Caenorhabditis briggsae. C. elegans TSP-17 is highlighted by a red box. (TIF) [file pgen.1004767.s002.tif]

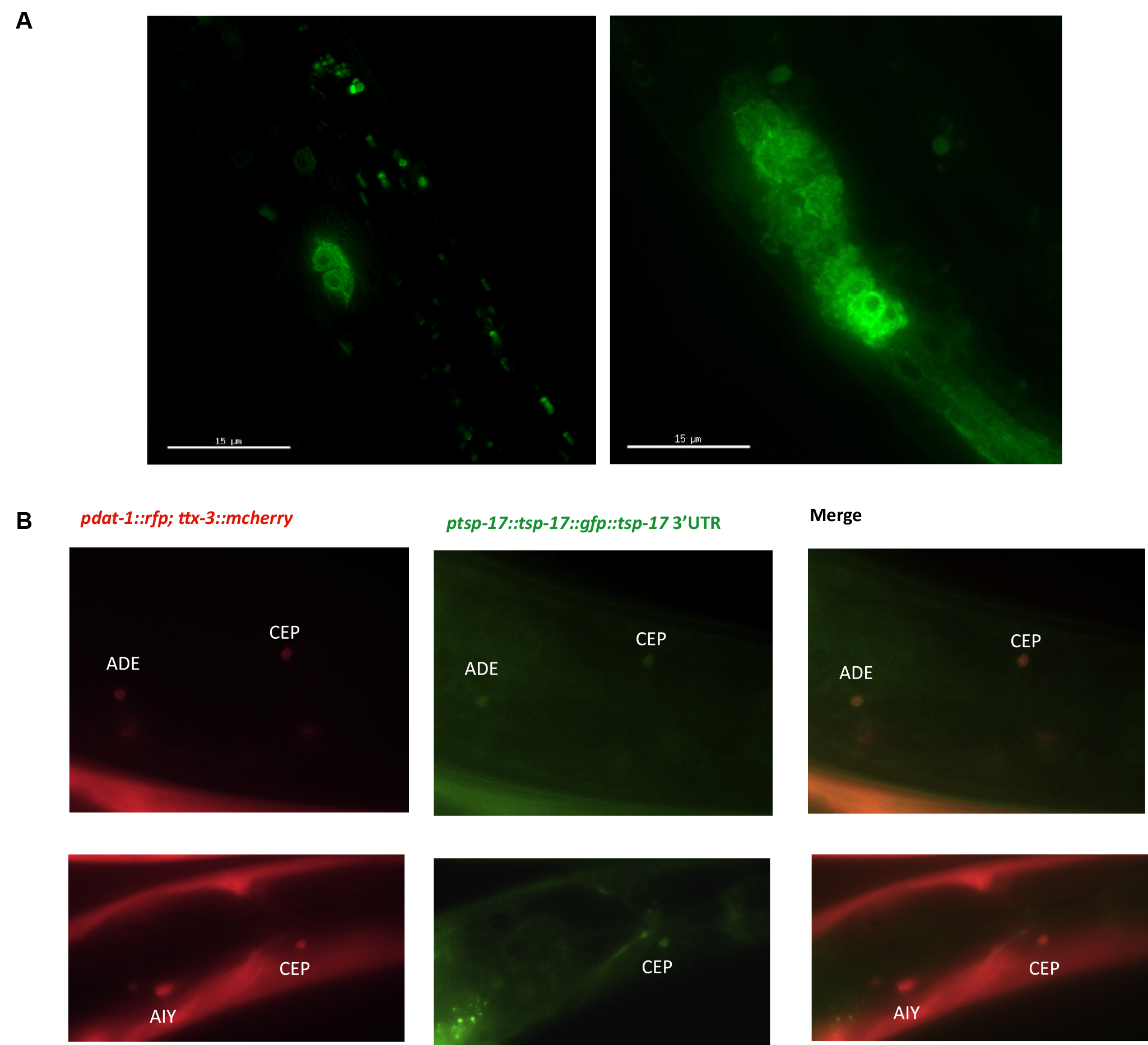

Supplement: Figure S3 — A. TSP-17(GT1681)::GFP expression in the vulva (left panel) and the spermatheca (right panel). Strain TG2474 was used. Images are projections of six Z-stacks. B. TSP-17 expression in ADE and CEP cell bodies. (TIF) [file pgen.1004767.s003.tif]

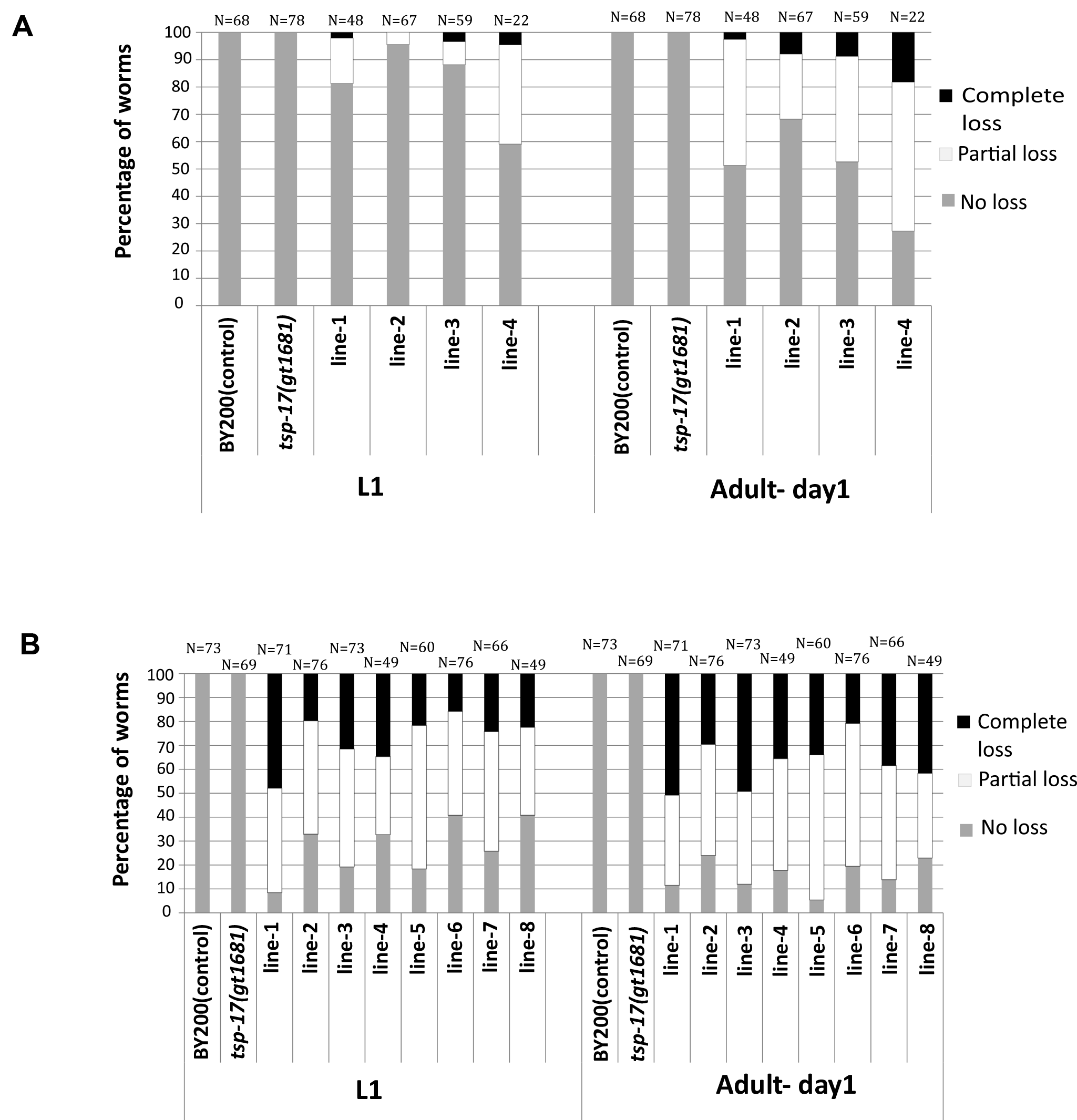

Supplement: Figure S4 — TSP-17 overexpression in a wild-type background induces neurodegeneration without 6-OHDA treatment. Strains used were (A) TG2440 for TSP-17 overexpression and (B) TG2474 TSP-17(gt1681) overexpression. (TIF) [file pgen.1004767.s004.tif]

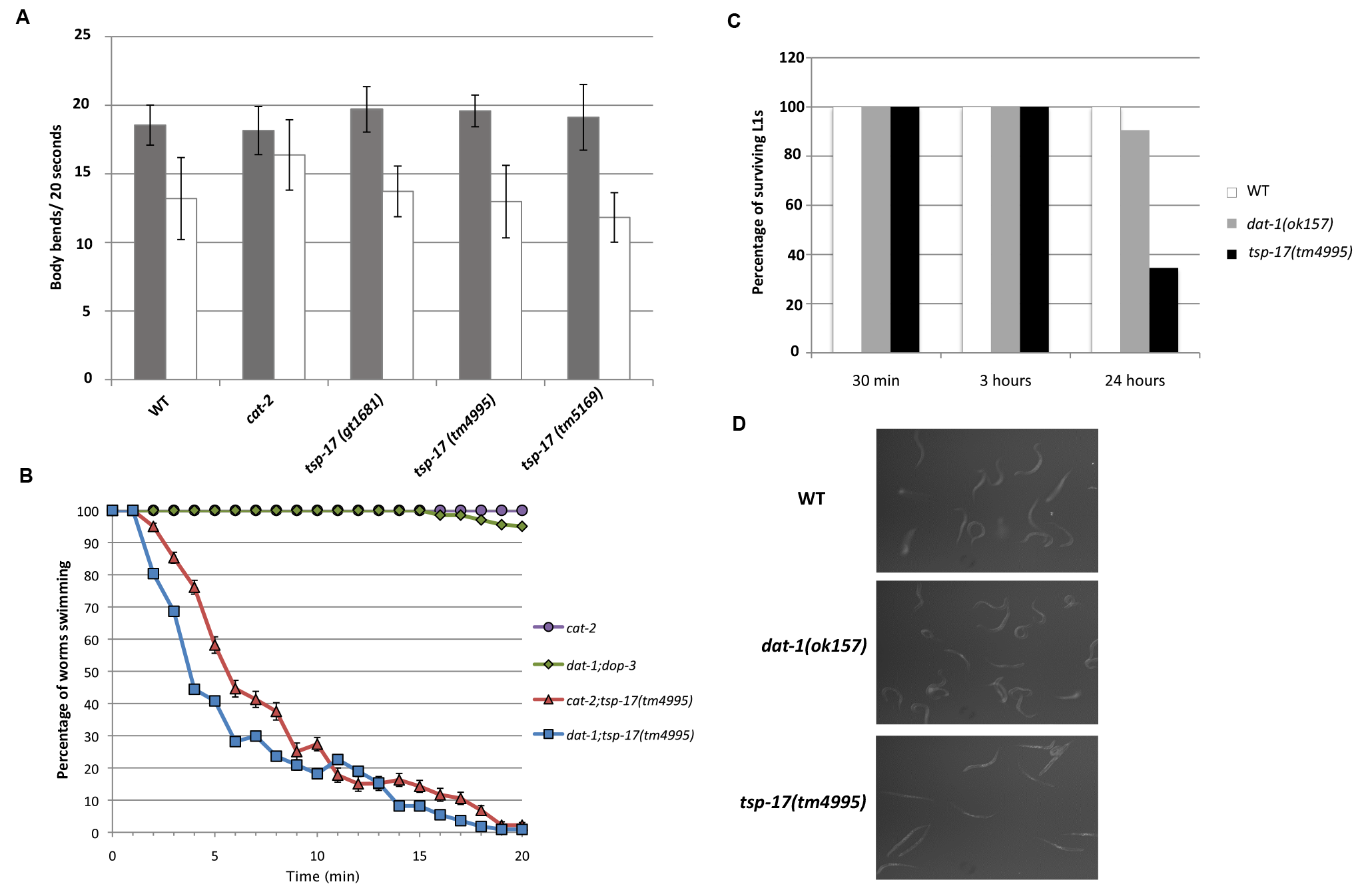

Supplement: Figure S5 — Analysis of tsp-17 behavioral phenotypes. A. Basal slowing response. Movement before (grey bars) and after reaching a lawn of bacteria (white bars) is indicated. B. Quantitative analysis of SWIP behavior in L1-stage worms, over 20 min. The SWIP phenotype of L1-stage tsp-17(tm4995) worms is not rescued by cat-2. Assays were done in triplicate. Error bars represent the standard error of the mean. C, D. L1 “swimming-induced lethality” phenotypes. Worms were incubated as for the L4 swimming induced paralysis assay and plated on seeded plates after the indicated times to assess viability. Representative pictures are shown in D. (TIF) [file pgen.1004767.s005.tif]

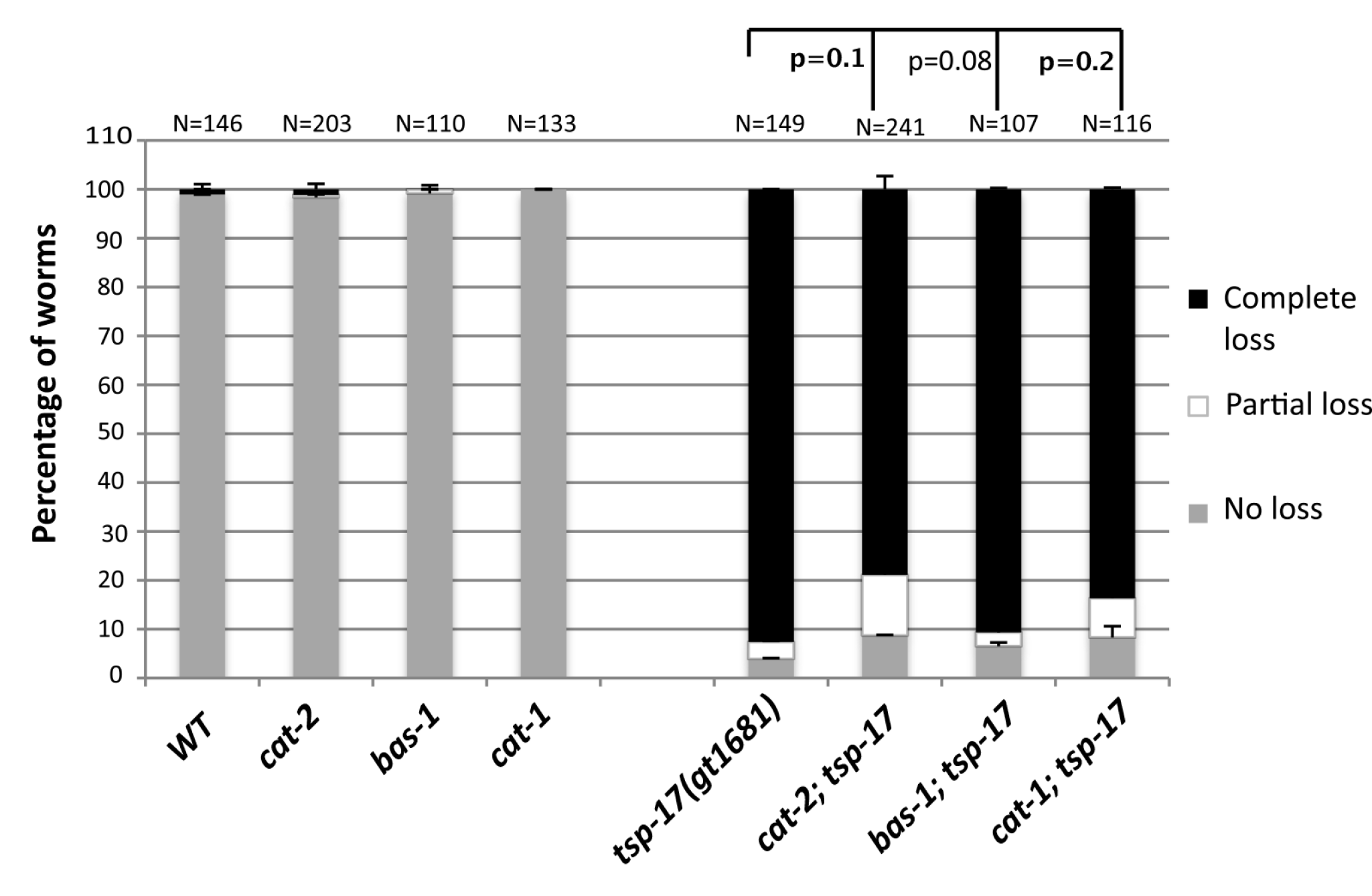

Supplement: Figure S6 — Analysis of 6-OHDA mediated neurodegeneration in cat-2 , bas-1 and cat-1 strains. Data presented is from scoring the extent of neurodegeneration 72 h post 6-OHDA intoxication. (TIF) [file pgen.1004767.s006.tif]

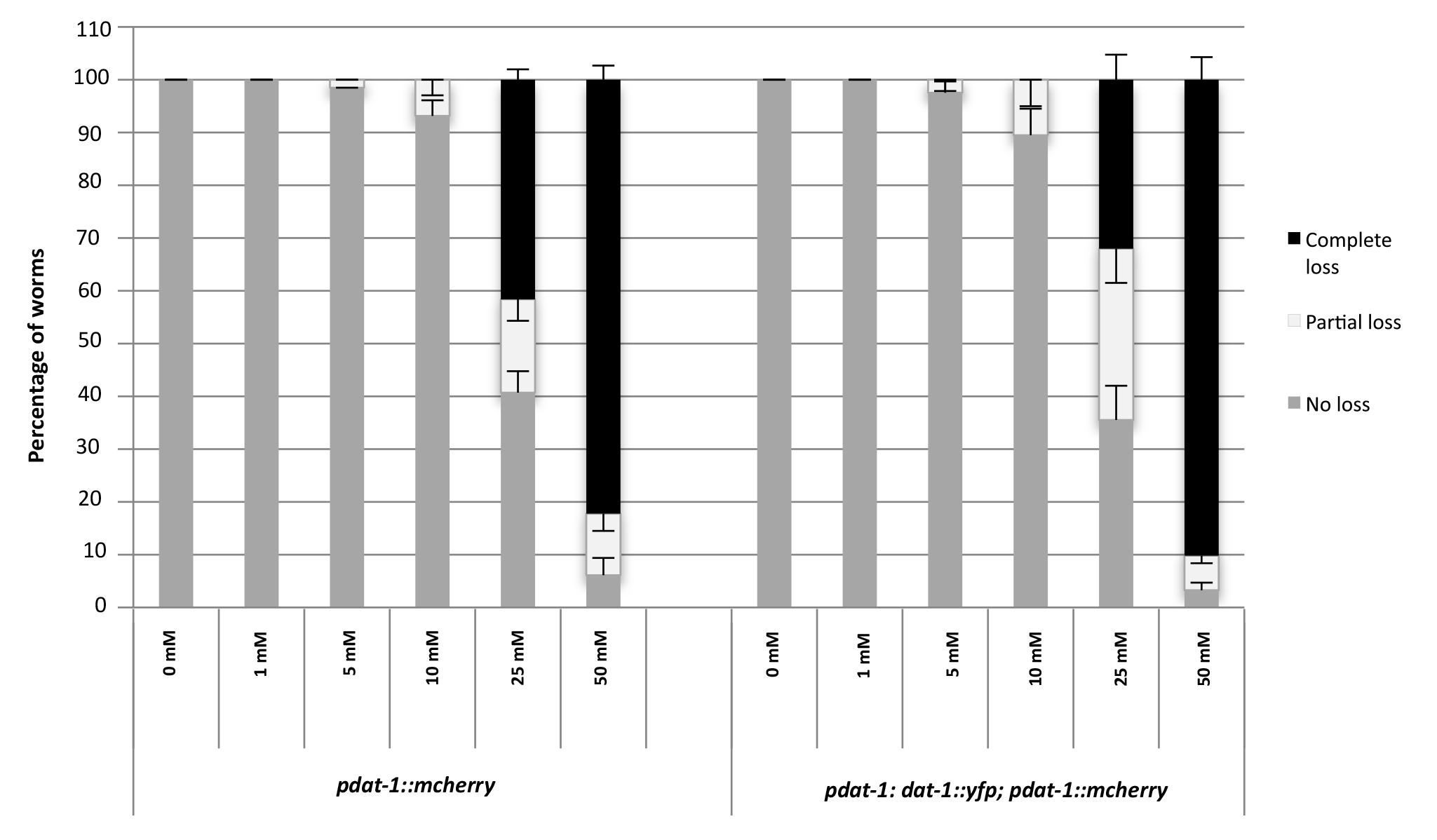

Supplement: Figure S7 — Dopamine receptors act antagonistically to modulate the 6-OHDA sensitivity of tsp-17(tm4995) mutants. Worms of the indicated genotypes were intoxicated with the indicated doses of 6-OHDA and scored 72 h after intoxication. Experiments were done in triplicate and the average data is presented. (TIF) [file pgen.1004767.s007.tif]

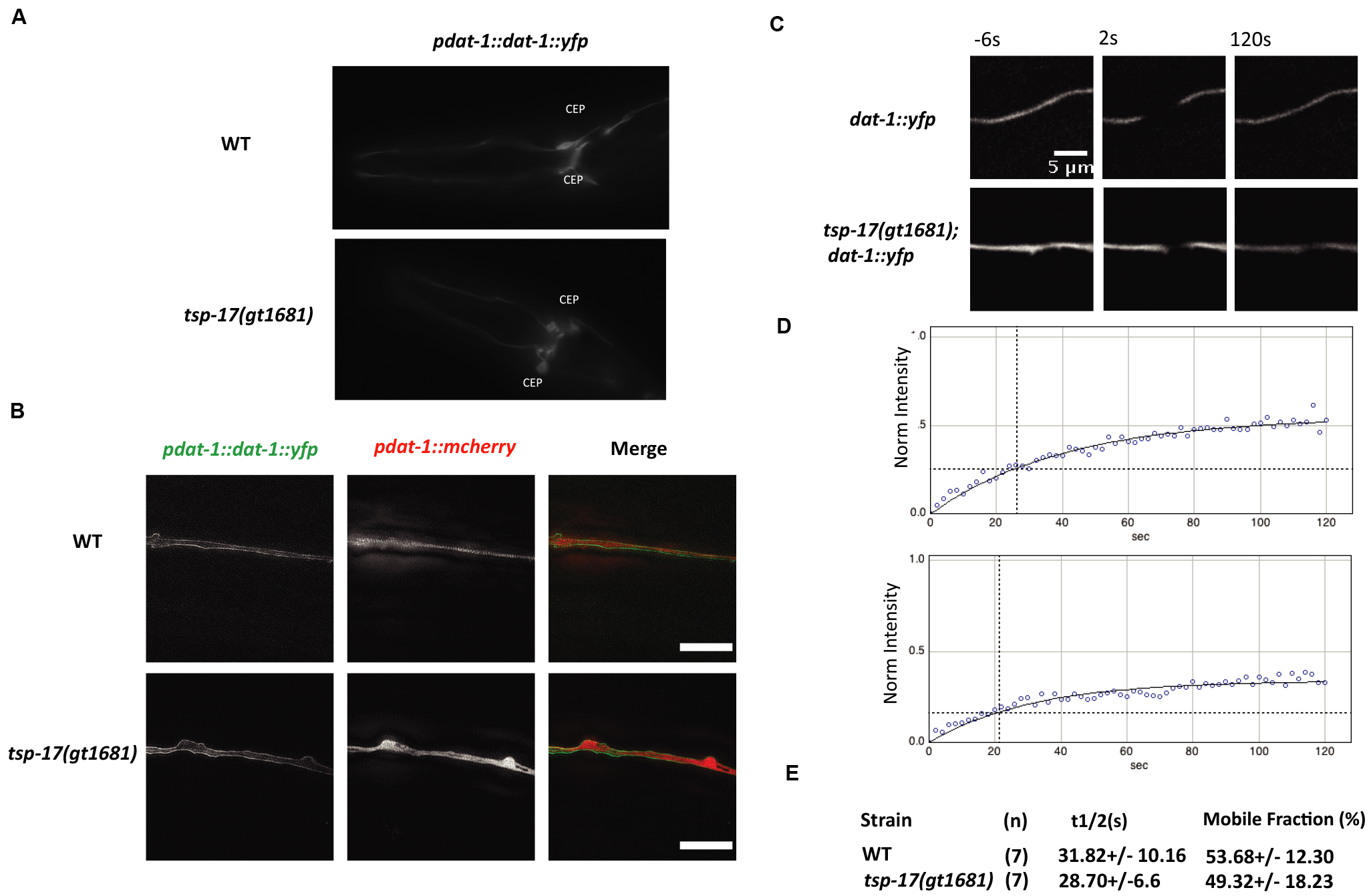

Supplement: Figure S8 — DAT-1::YFP expression and half live is not altered in tsp-17(gt1681) mutant worms. A. Expression of TSP-17 in CEP neurons in wild-type (TG2470) and tsp-17(gt1681) mutants (TG2471). B. Structural Illumination ‘super resolution’ images of a CEP dendrite in wild-type and tsp-17(gt1681) worms showing membrane localization of DAT-1::YFP relative to a cytoplasmic mCherry marker. There are no differences in expression. The crosshatching-like pattern is an artifact introduced by the diffraction grid used in acquisition, not a feature of expression. Scale bar (white) is 5 µm in length. Images are 18 µm×18 µm. C. Representative FRAP images of DAT-1::YFP taken prior to bleaching (−6 s), immediately after the bleach event (2 s) and after 2 minutes post bleaching (120 s). Images are 18 µm×18 µm. D. Representative graphs showing normalized recovery curves in wild-type (top) and tsp-17(gt1681) (bottom) worms. Example half time of recovery (t1/2) for each graph is shown at the intersection of the dashed lines. The mobile fraction is the point at which the curve plateaus. E. Average values and standard deviation for t1/2 and mobile fractions for DAT-1::YFP wild-type and tsp-17(gt1681) worms (n = 7). (TIF) [file pgen.1004767.s008.tif]

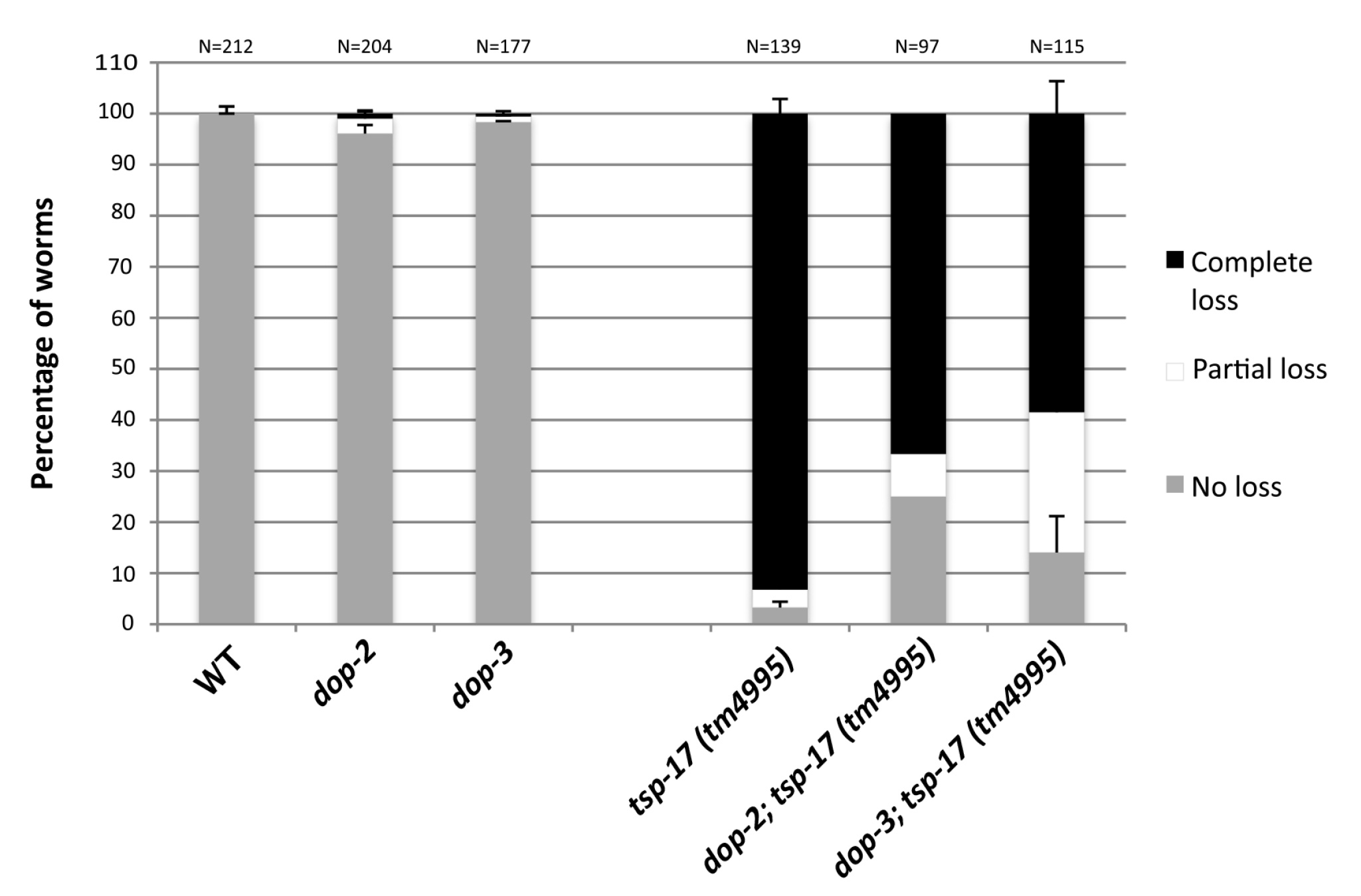

Supplement: Figure S9 — Dopamine receptors act antagonistically to modulate the 6-OHDA sensitivity of tsp-17(tm4995) mutants. Worms of the indicated genotypes were intoxicated with 10 mM 6-OHDA and scored 72 h after intoxication. Experiments were done in triplicate and the average data is presented. (TIF) [file pgen.1004767.s009.tif]
